# A Beta-Herpesvirus with Fluorescent Capsids to Study Transport in Living Cells

**DOI:** 10.1371/journal.pone.0040585

**Published:** 2012-07-11

**Authors:** Jens B. Bosse, Rudolf Bauerfeind, Leonhard Popilka, Lisa Marcinowski, Martina Taeglich, Christophe Jung, Hannah Striebinger, Jens von Einem, Ulrike Gaul, Paul Walther, Ulrich H. Koszinowski, Zsolt Ruzsics

**Affiliations:** 1 Max von Pettenkofer-Institute, Ludwig Maximilians University, Munich, Germany; 2 Department of Cell Biology, Hannover Medical School, Hannover, Germany; 3 Institute of Virology, University Medical Center Ulm, Ulm, Germany; 4 Department of Biochemistry, Gene Center, Ludwig Maximilians University, Munich, Germany; 5 Central Unit for Electron Microscopy, University of Ulm, Ulm, Germany; University of Edinburgh, United Kingdom

## Abstract

Fluorescent tagging of viral particles by genetic means enables the study of virus dynamics in living cells. However, the study of beta-herpesvirus entry and morphogenesis by this method is currently limited. This is due to the lack of replication competent, capsid-tagged fluorescent viruses. Here, we report on viable recombinant MCMVs carrying ectopic insertions of the small capsid protein (SCP) fused to fluorescent proteins (FPs). The FPs were inserted into an internal position which allowed the production of viable, fluorescently labeled cytomegaloviruses, which replicated with wild type kinetics in cell culture. Fluorescent particles were readily detectable by several methods. Moreover, in a spread assay, labeled capsids accumulated around the nucleus of the newly infected cells without any detectable viral gene expression suggesting normal entry and particle trafficking. These recombinants were used to record particle dynamics by live-cell microscopy during MCMV egress with high spatial as well as temporal resolution. From the resulting tracks we obtained not only mean track velocities but also their mean square displacements and diffusion coefficients. With this key information, we were able to describe particle behavior at high detail and discriminate between particle tracks exhibiting directed movement and tracks in which particles exhibited free or anomalous diffusion.

## Introduction

Herpesviruses are doublestranded DNA viruses which seem to have coevolved with their respective hosts [1]. Currently, eight different human herpesviruses are known. Three can be grouped into the alpha-subfamily (herpes simplex virus 1, HSV-1; herpes simplex virus 2, HSV-2; varicella zoster virus, VZV), three into the beta-subfamily (human cytomegalovirus, HCMV; human herpes virus 6, HHV-6; human herpes virus 7, HHV-7) and two into the gamma-subfamily (Epstein-Barr virus, EBV; Karposi-sarcoma associated virus, KSHV). Despite clinical importance of all human herpesviruses, most pioneering basic research is done studying alpha-herpesviruses. It is, however, not clear to what extend results obtained in one subfamily can be assigned to the other herpesviruses as comparative studies of the herpesvirus lytic life cycle are only possible since the introduction of reverse genetics into all subfamilies [2], [3], [4], [5].

One of the less understood aspects of viral morphogenesis are the dynamics of viral particle transport. These dynamic events are studied best by fluorescently labeled virus particles in combination with live cell fluorescence microscopy. In recent years, recombinant viruses expressing fusions of structural proteins to fluorescent proteins widely extended our understanding of the dynamic processes involved in the morphogenesis of a large number of different viruses [6], [7], [8], [9], [10], [11], [12], [13], [14], [15], [16].

The reported approaches concerning herpesviruses can be roughly divided into three groups. The first approach is the labeling of a glycoprotein. This allows the tracking of the first steps of infection from attachment to fusion with host membranes, or from envelopment to release [17], [18]. The second approach is the utilization of a tegument protein as fusion partner [15], [19], [20]. Depending on the protein used, this approach allows the tracing of events after fusion and before envelopment and even in the host nucleus after attachment of the fusion product to the capsid. The last approach utilizes a capsid protein [7], [21], [22]. Only this approach allows the tracing of most steps of viral morphogenesis except the ones that solely concern the processing of viral DNA. Though, a combination of these approaches [5], [23], [24], [25], [26] can be useful to discern morphogenesis steps, e.g. enveloped from non-enveloped capsids. A capsid-tagged recombinant virus is therefore most desirable as it allows the tracing of almost all morphogenesis steps. Until now however, only alpha-herpesviruses like HSV-1, pseudorabies virus (PRV) and equine herpes virus type 1 (EHV-1) [7], [22], [27], [28], [29], [30] could be engineered to express fluorescent capsids. In contrast, all attempts to fuse fluorescent proteins to beta- or gamma-herpesviruses capsid proteins failed so far [31], [32], [33].

We therefore aimed to establish new recombinant beta-herpesviruses expressing labeled capsids by fusing a fluorescent protein (FP) to a capsid protein. This is however, not an easy task. Most herpesvirus capsid proteins undergo numerous and highly ordered interactions with themselves or other proteins to build the capsid [32]. The pure bulk of several copies of FPs that are needed to deliver a bright fluorescent signal induces sterical problems and may render the recombinant viruses non-viable. As first described by Desai et al. [7] the small capsid protein (SCP) of some alpha-herpesviruses like HSV-1, PRV [22] VZV [21] and EHV [27] seem to tolerate an aminoterminal fusion to FPs. This capsid protein is exceptionally suitable as a fusion partner as it is located at the outermost of the core-capsid structure, which apparently gives sufficient steric freedom to accept a fusion to bulky fluorescent proteins. Moreover, it is a very abundant protein with 900 copies per capsid (6 copies per hexon), which results in a sufficiently bright fluorescent signal if fused to a fluorescent protein.

Yet, the tagging of the SCP in HSV-1 resulted in a moderate growth defect with titers reduced to about 50% compared to wild type virus. Moreover, the extent of attenuation was indistinguishable from a SCP null mutant [5] arguing for the notion that GFP-tagging of some SCPs rendered them biologically inactive. This is further supported by another study on PRV showing that the aminoterminal fusion with GFP renders the fusion product biologically non-functional [34]. It emerges therefore, that the tagging of alpha-herpesvirus SCPs is apparently only possible because they are not essential for virus replication.

In contrast to alpha-herpesviruses the SCPs of beta- as well as gamma-herpesviruses are essential and all reported attempts to fuse a fluorescent protein to SCPs of these subfamilies resulted in non-viable or even dominant negative mutants [31], [32], [33], [35].

Here, we report on the first successful construction of fluorescent, capsid-tagged murine cytomegaloviruses (MCMV). These viable viruses carried ectopic insertions of SCP-FP fusions leaving the wild type (WT) locus of the respective SCP gene untouched. The resulting recombinant viruses were viable and exhibited bright fluorescence signals. We used these recombinant viruses to study capsid transport both during virus entry and egress by single particle tracking. We could show that intracellular transport of MCMV-particles is mostly directed and dependent of microtubules.

## Materials and Methods

### Cells and Viruses

BALB/c murine embryonic fibroblasts (MEFs), M2-10B4 bone marrow stromal cells (ATCC CRL-1972), and NIH-3T3 fibroblasts (ATCC CRL-1658) were prepared and treated as described previously [36]. All MCMVs used in this study are based on the pSM3fr-Δm1-16-FRT bacterial artificial chromosome (BAC) [37]. The corresponding virus designated MCMV-Δm1-16-FRT, lacking the leftmost 16 genes which encode for viral immunoevasins [38], is designated WT throughout this work because its replication properties were extensively studied recently and was found to be indistinguishable from the wild type virus in tissue culture [37]. All viruses reported in this study were reconstituted from their respective BACs by nucleofection of NIH/3T3 cells. Nucleofection was done using an Amaxa 96-Well Shuttle system (Lonza) according to the manufacturers’ instructions for NIH-3T3 cells. In brief, 5×10^5^ cells were nucleofected with 400 ng purified BAC-DNA and seeded together with 1.5×10^5^ non-transfected cells into one well of a 24 well plate. 3 days after nucleofection, the cell layer was checked for plaques and split 1∶4. Usually, six days after nucleofection, full CPE could be observed. Cultures were harvested and samples were frozen as passage zero. Afterwards, virus inoculi were scaled up on M2-10B4 cells as described previously [36]. Virus titers were quantified on MEFs by a standard plaque assay [39].

### Analysis of Viral Growth in vitro

MEFs were infected with the viruses to be analyzed in duplicate at a multiplicity of infection (MOI) of 0.1 plaque forming unit (PFU) per cell at 37^o^C. The inoculi were removed after 1 h, then the cultures were washed three times with Dulbeccós phosphate-buffered saline (DPBS), normal medium was added and the incubation was continued. Supernatants of infected cells were harvested daily on days 1 to 5 after infection, and the amounts of the released infectious particles were determined by plaque assay on MEFs.

To determine plaque sizes, standard plaque assays on MEF were performed in 48-well plates and infected cultures were overlaid with carboxyl-methylcellulose as described previously [39]. 5 days post infection the overlay was removed and standard immunostaining (see below) was performed with a monoclonal antibody against MCMV ie-1 [58] and matching Alexa-conjugated secondary antibodies (Invitrogen). Digital images of individual plaques were obtained by confocal microscopy (see below) and their pixel size was measured using ImageJs line tool [40] and converted into µm.

### Plasmid Construction

To generate the pOTO-S-GFP-SCP construct we amplified a sequence with primers SCPPfor and SCPPrev (Table S1) binding to positions 74153–74173 and 73766–73786 of the MCMV Smith strain (NCBI Reference Sequence: NC_004065.1), respectively. The amplicon coded for a proposed m48.2 promoter sequence as well as the first 34 amino acids (aa) of m48.2. The primer SCPPfor added an *Eco*RV site to the 5′ end while SCPPrev fused *Afl*II and *Spe*I sites as well as a hemagglutinin (HA)-tag coding sequence to the 3′ end. The PCR fragment was cut with *Eco*RV and *Afl*II and inserted into the previously described plasmid pO6-IET-gfpSCP [33] replacing its *Afl*II -*Ssp*I fragment.

A further variant of this rescue vector coding for a SCP fusion to mCherry was generated by amplifying the respective fluorescent proteins with primers SP-*Afl*II-FP-*Bsr*GI and ASP-*Afl*II-FP-*Bsr*GI (Table S1) from pmCherry-C1 (Clontech) which added *Afl*II and *Bsr*GI sites to the respective ends. The PCR-products were cut with *Bsr*GI and *Afl*II and and inserted into the previously described plasmid pOTO-S-GFP-SCP, replacing its *Bsr*GI-*Afl*II fragment resulting in pOTO-S-mCherry-SCP, respectively.

To generate the S-GFP-SCP* mutant, lacking the last 14 C-terminal codons of the SCP open reading frame (ORF), the parental vector pOTO-S-GFP-SCP was cut with *Pvu*II and *Apa*I thereby removing the coding sequence for the last 17 aa of the C-terminal region of m48.2. To subsequently restore the missing nine codons as well as the stop codon, the corresponding region pOTO-S-GFP-SCP was amplified with primers SP-MCP and ASP-MCP (Table S1) and inserted by *Pvu*II and *Apa*I resulting in pOTO-S-GFP-SCP*.

Recombinational cloning to generate yeast bait and prey vectors for yeast two hybrid tests was essentially done as described in Fossum et al. [41]. The plasmids pOTO-S-GFP-SCP or pOTO-S-GFP-SCP were used as PCR templates for insert amplification with primers attB1-S-GFP-SCP-attB2-SP, attB1-S-GFP-SCP-binding-negative-No-Stop-attB2-ASP and attB1-SCP-S-GFP-SCP-no-stop-attB2-ASP (Table S1). Resulting PCR products were subjected to BP-recombination by Gateway-mediated recombination into pDONR-221 according to the manufacturer’s instructions (Invitrogen). Subsequently, the coding regions were transferred to the prey plasmid pGADCg [42], generating the plasmids pGADCg-S-GFP-SCP and pGADCg-S-GFP-SCP* which code for a C-terminal fusion to the activation domain (AD).

The integrity of all constructs mentioned above, was confirmed by analytical digests and DNA sequencing of crucial elements.

### Construction of Recombinant MCMV BACs

To insert the expression cassettes into the MCMV BAC we inserted the above described rescue plasmids into a MCMV-BAC by the FRT/Flp system. Escherichia coli strain DH10B (Invitrogen) containing the pSM3fr-Δm1-16-FRT BAC [37] and the temperature-sensitive Flp recombinase expression plasmid pCP20 [43] was transformed with various pOTO-constructs carrying the different SCP fusions and treated as described previously [33]. Correct recombinations were verified by restriction analysis and sequencing *in loco*.

### Yeast Two Hybrid Analysis

Yeast two-hybrid analysis of interactions between SCP constructs and MCP was performed as described in the manufacturer’s protocol (BD Biosciences/Clontech). Briefly, the bait plasmid pGBKT7-MCP [41] was transformed into the yeast strain Y187 while the prey plasmids pGADCg-S-GFP-SCP and pGADCg-S-GFP-SCP* were transformed into the AH109 strain. Diploid yeast cells carrying both vectors were generated by mating the strains and subsequent selection on SDC-Leu-Trp medium. Growth of diploid cells on SDC-Leu-Trp-His medium was used to assess bait-pray interaction via the HIS3 reporter. Bait and prey plasmids without inserts were used as a control.

### Density Gradient Purification of Nuclear and Extracellular MCMV Particles

Gradient purification of extracellular MCMV particles was either done on self-forming Optiprep (Sigma) gradients or with 10–40% preformed Nycodenz (Axis-Shield). In both cases supernatants from virus-infected cells were centrifuged at low speed (5500 g/15 min) to remove cell debris. Afterwards, virions were concentrated by high-speed centrifugation (23000 g/210 min) and the resulting virus pellet was carefully resuspended in VS-buffer (0.05 M Tris, 0.012 M KCL, 0.005 M EDTA, pH 7.8). Free DNA/RNA was removed by overnight treatment with 625 U/ml *Benzonase* (Novagen) at 4°C.

Nycodenz-gradient purification was essentially done as described in Doehner et al. [44]. In brief, the resulting suspension was loaded onto a continuous 10–40% Nycodenz (Axis-Shield) density gradient prepared on a *GradientStation ip* (Biocomp) and separated at 20000 g for 105 min in a Beckman SW28 rotor at 4°C. The resulting bands were visualized and collected on a *GradientStation ip*.

Purification of particles in self-forming Optiprep gradients was done by mixing equal amounts of a 60% Optiprep solution and resuspended pellet. The mixture was then loaded into centrifuge tubes and spun at 144.000 g for 16 h. Resulting bands were visualized over a light source and collected.

Sucrose-gradient purification of nuclear MCMV particles was done as described by Radtke et al. [45]. After density purification on a sucrose gradient, bands were collected as described above. All fractions were analyzed by standard negative-stain electron microscopy (EM) [46] verifying their integrity and purity.

### Analytical Polymerase Chain Reactions (PCRs)

Viral DNA was extracted from Benzonase (Novagen) treated, gradient purified virions using the DNeasy blood and tissue kit (Qiagen) according to the manufacturer’s instructions. It was quantified by real-time PCR using an ABI Prism 7700 sequence detector (Applied Biosystems). Prior to amplification, purified DNA was digested with *Pae*I for 1 h, 37°C followed by heat inactivation. To determine the ratio of viral genome copy number to PFU, a quantitative PCR was performed for each sample in triplicate using MCMV M54 specific primers and probes as described by Scrivano et al. [47]. Viral DNA copy numbers were then calculated by comparing the amplification to a standard curve generated by using a pSM3fr-BAC DNA template. To normalize for loss of DNA during purification, we determined the ratio of a defined quantity of pSM3fr-BAC DNA before and after purification.

### Immunoblotting

Viral protein expression was analyzed by infecting a confluent layer of M2-10B4 cells at a MOI of 1 for 48 h in 6 cm dishes. Subsequently, cells were washed with DPBS and lysed in total lysis buffer (62.5 mM Tris, pH 6.8, 2% (v/v) sodium dodecyl sulfate (SDS), 10% (v/v) glycerol, 6 M urea, 5% (v/v) β-mercaptoethanol, 0.01% (w/v) bromophenol blue, 0.01% (w/vol) phenol red) plus 125 U of *Benzonase* (Novagen) for 90 min at 4°C. After denaturation at 95°C for 10 min, samples were loaded onto 15% SDS-polyacrylamide gels and separated by electrophoresis (SDS-PAGE).

To analyze proteins from purified virions, a volume corresponding to approximately 1.1×10^5^ PFU of gradient-purified virus preparation per lane was diluted in 10 ml of TNE buffer (100 mM NaCl, 10 mM EDTA, 50 mM Tris-Cl pH 7.5). Virus particles were precipitated by spinning the samples at 107.000 g for 1 h at 4° C. The supernatant was discarded and the pellet resuspended in reducing 1× LDS buffer (Invitrogen), loaded onto 4–15% Bis-Tris gels (Invitrogen) and separated by SDS-PAGE.

After SDS-PAGE, separated proteins were transferred onto *PVDF* membranes in the presence of blotting buffer (25 mM Tris, 192 mM glycine, 20% (v/v)] methanol, pH 8.3). Membranes were blocked in TBS-T (Tris- buffered saline, 0.05% (v/v) Tween 20) containing 5% BSA or 5% dry milk for 1 h at room temperature (RT). To detect the SCP of MCMV, a polyclonal antiserum directed against SCP was generated by immunizing rabbits with Ovalbumin (OVA) -coupled peptides corresponding to aa 36–50 and 59–86 m48.2 ORF (Metabion). To detect MCP, a rat polyclonal antiserum was used [33]. GFP was detected by a polyclonal rabbit antiserum (Invitrogen). mCherry was detected with a polyclonal rabbit antiserum generously provided by Vincent Geli (CNRS Marseille) through Zemer Gitai (Princeton University). The blocked membranes were incubated with the respective antisera overnight at 4°C and subsequently washed with TBS-T and incubated with the appropriate horseradish peroxidase-conjugated (POX) secondary antibody. The HA-tag was detected with a HA-specific, POX-conjugated monoclonal rat antibody (Roche) according to the manufacturer instructions. The Western blot reactions were visualized with SuperSignal West Dura Extended Duration Substrate (Pierce) using a Fusion luminiscence reader (Vilber) or autoradiography film and scanned afterwards. Western blot images were slightly sharpened afterwards with the unsharp mask tool in Adobe Photoshop.

### Immobilization of Virions on Glass Coverslips

Immobilization of MCMV particles was performed by loading approximately 1×10^5^ PFU of density gradient-purified virus preparations diluted in 200 µl DBPS onto fibronectin-coated glass coverslips and incubated for 1 h at 37°C. After binding, virions were subjected to immunostaining as described below.

### Immunostaining for Microscopy

Indirect immunofluorescence analysis was carried out on glass slides, channel- or 8 well µ-slides (Ibidi) by fixing cells or virions with 4% paraformaldehyde in DPBS (w/v) for 10 min at 37°C. The fixative solution was replenished twice with DPBS and the cells were permeabilized for 10 minutes with a solution of 0.1% Triton X-100 in DPBS. After extensive washing with DPBS, the cells were blocked using 3% (w/v) BSA in DPBS (blocking solution) for 1 h at RT. Primary antibodies (anti-MCP rat polyclonal antiserum [33], anti-GFP rabbit polyclonal antiserum (Invitrogen), anti-HA-tag rat monoclonal rat antibody (Roche)) were applied in blocking solution and incubated with the cells or virions at RT for 1 h followed by three DPBS washing steps and 1 h incubation at RT with 1∶1000 dilutions of Alexa Fluor-conjugated, specific secondary antibodies (Invitrogen) in blocking solution. After a final extensive washing step with DPBS, the preparations were imaged directly or mounted on glass slides with Prolong Gold (Invitrogen).

### Quantification of Particle Fluorescence Intensity Distributions

About 1×10^4^ PFU of gradient purified virus preparations diluted in 400 µl DPBS were bound on Poly-Lysin coated glass-bottom dishes (MatTek) for 1 h at 4°C in the dark. Afterwards dishes were rinsed once with DPBS and directly imaged on a Nikon epifluorescence microscope equipped with a 60× NA 1.4 objective. Pictures were recorded in 16 bit mode with an EMCCD camera (Andor iXon 3) and exposure times as well as the EM gain were adjusted to maximize the recorded dynamic range.

Image analysis was carried out in Matlab 2011b using a custom-designed software package. The routines loaded each saved image, removed a slowly-varying background, located the brightest objects (those greater than 2.5 times the standard deviation of the background) in each image, extracted a region of interest about each candidate object, then fitted each candidate to a 2D Gaussian profile. Candidate source locations were discriminated from spurious events not consistent with a 2D model of the point spread function by selectively cutting on the goodness of fit (chi square) and the width of the fit. The remaining data informed about the fluorescence distributions of the source population. After initial isolation and selection, remaining particles were used to determine the fluorescence distribution of the population by integrating the best fit Gaussian model over the region of interest for each particle.

### Fluorescence Microscopy

Confocal laser scanning microscopy was performed with either a Zeiss LSM510 Meta system, a LSM710 system or an Olympus FV1000 system equipped with high numerical aperture oil immersion objectives. GFP was excited with a 488 nm laser line. mCherry was excited with a 543 nm or 561 nm laser line. Protein fluorescence emission was detected using corresponding filter sets of the microscopes. Alexa Fluor-Dyes were detected corresponding to the manufacturers’ instructions. Pinhole diameters were adjusted to a width corresponding to an optical slice thickness of 1 µm for all channels. Pixel sizes were chosen based on the Nyquist criteria and varied around 110 nm/Pixel in case of a 63x, NA 1.4 objective. In general, imaging conditions were chosen which resulted in an optimal compromise between light exposure and detector noise and were kept constant in between acquisitions of the same experiment. Live-cell imaging was performed with cells growing in µ-slides (Ibidi) or glass-bottom dishes (MatTek). During acquisition, the cells were incubated in an environmentally controlled chamber at 37°C with either 5% CO_2_ or in CO_2_-independent medium (Invitrogen). All images were processed according to the journals guidelines using ImageJ [40] as well as Photoshop CS4 (Adobe) software. Specially, noise in time-lapse stacks was reduced by Kalman filtering in ImageJ. Gradial bleaching was corrected by using the ImageJ Bleach Correction plugin (written by J. Riedorf). All images were contrast-enhanced to optimize for printing.

### Single Particle Tracking

The single virus particle fluorescence patterns obtained were fitted by a two-dimensional Gaussian function:
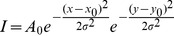
(1)where A_0_ and σ are the amplitude and the width at half-maximum of the two-dimensional Gaussian curve, and x_0_ and y_0_ the coordinates of the position of the individual virus particles. The single particle tracking procedure was automated using a custom-written Labview software (National Instruments) [48].

For each trajectory, a set of values for the square displacement, r^2^(t), between two observations separated by the time lag t_lag_  =  n • Δt (where Δt is the time interval between successive frames of the movies, n  = 0, 1, 2, …, N-1 with N being the total number of points in a trajectory) was computed according to:
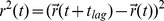
(2)


The Mean Square Displacements (MSD =  <r^2^(t)> ) were then plotted for every time lag t_lag_ and the MSD plots were fitted for the 10% first time lags (10 points) assuming three different modes of possible motion [49]:

Normal diffusion:
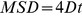
(3)


Anomalous diffusion:
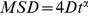
(4)


Directed motion with diffusion:
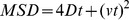
(5)where D is the diffusion coefficient, α the anomalous exponent and *v* the velocity of the directed transport.

To determine for each single trajectory which mode of motion described the data best, each MSD plot was fitted by Equations 3, 4 and 5 and the reduced chi-squared of the fits were compared. The normal diffusion model (Equation 3) was used as long as the more complex model functions of for anomalous diffusion (Equation 4) and directed motion with diffusion (Equation 5) did not resulted in a at least two-fold decrease of the reduced chi-squared of the fit. In cases in which this condition was fulfilled, the mode of motion with the minimum reduced chi-squared was chosen. Hence, this procedure leads to the clustering of diffusing particles into three distinct classes representing different modes of diffusion.

### Transmission Electron Microscopy (EM)

NIH 3T3 or M2-10B4 cells were grown on carbon-coated sapphire discs and infected at a MOI of 0.5 with centrifugal enhancement at 1600 g/RT for 30 min. After an additional hour, the inoculum was replaced by normal medium and cells were incubated for 48 h. Then, cells were fixed by high-pressure freezing with an *HPF 01* instrument (M. Wohlwend GmbH), freeze-substituted, and plastic embedded as described previously [50]. Embedded samples were thin-sectioned and viewed on a Zeiss EM 10 at 80 kV in transmission mode. The phenotype of infected NIH 3T3 or M2-10B4 cells did not differ. Pictures were recorded on EM film (MACO), digitalized at 1200 dpi, contrast-enhanced and sharpened with an unsharp mask according to the journals guidelines in Photoshop CS4 (Adobe).

### Immunoelectron Microscopy

Nuclear capsids were adsorpted onto carbon and Formvar-film coated 400 mesh copper grids (Stork Veco), the samples were washed with PBS. After blocking unspecific protein binding sites with 10 mg/ml BSA in PBS the grids were incubated for 30 min with rabbit GFP-antiserum (Invitrogen, dilution 1∶100), then washed with PBS followed by a 15 min incubation with protein-A gold (10 nm; Cell Microscopy Center, Utrecht School of Medicine, The Netherlands). After washing with PBS and distilled water, preparations were negatively contrasted using 2% (w/v) uranyl acetate (Merck), and analyzed with a Tecnai G20 (FEI) at 200 kV. For quantitation images of capsids were randomly taken at a magnification of 55,000x.

## Results

### Construction of Recombinant MCMVs Producing Fluorescent Capsids

**Figure 1:**
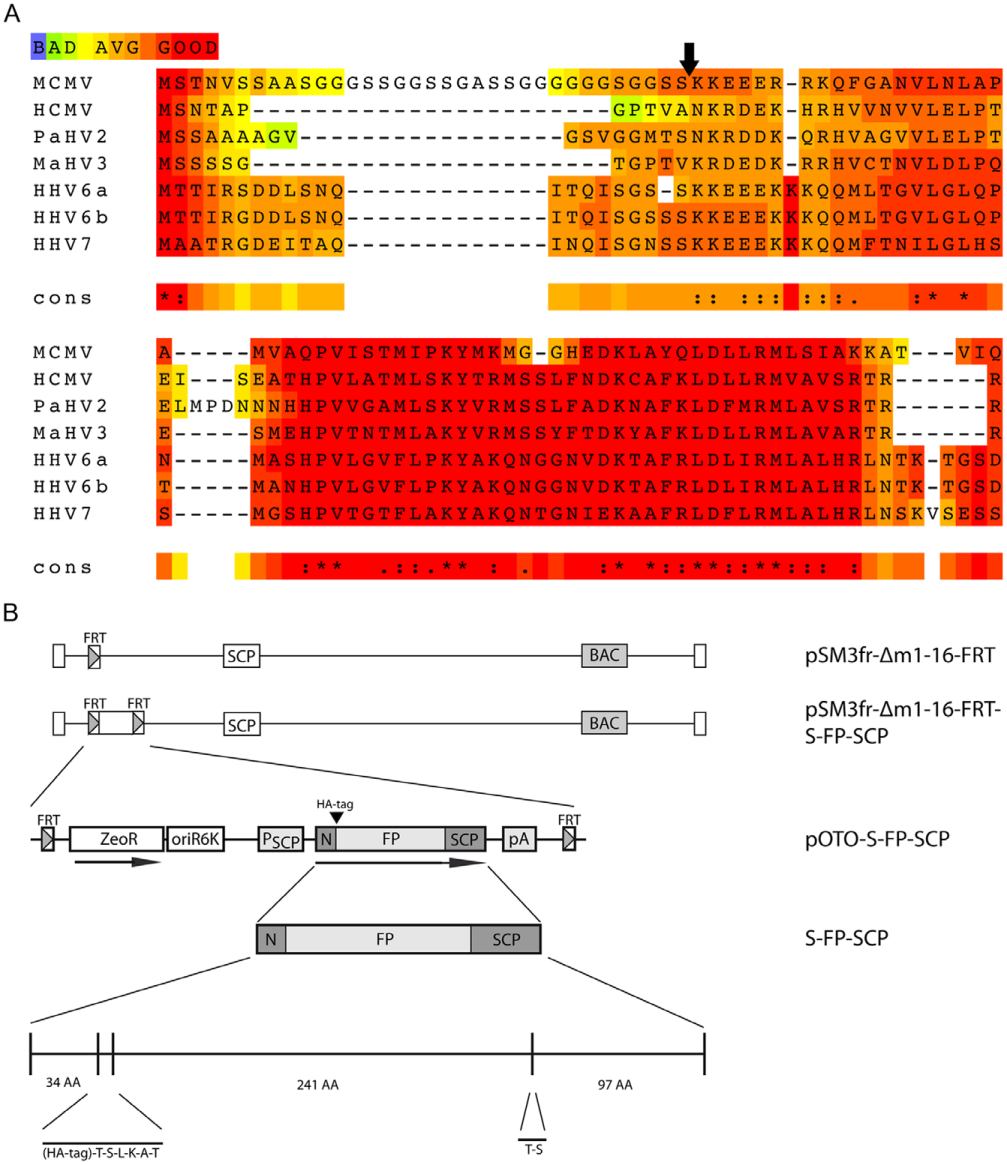
Construction of SCP fusion proteins. (A) Alignment of beta-herpesvirus SCP sequences with T-Coffee [62]. A naturally occurring glycin-serin-linker like sequence separates the N-terminus in MCMV. (B) Detailed representation of the S-GFP-SCP fusion protein as well as the basic genetic layout of the used mutant viruses carrying a fluorescent protein (FP) and a hemagglutinin tag (HA). GFP or mCherry were used as FPs. The FP could be removed by a simple SpeI-mediated digest resulting in a construct encoding for just a HA-tagged SCP. N marks the duplicated N-terminal region of SCP. The plasmids carrying the fusion constructs were inserted into the MCMV BAC by Flp-mediated recombination.

Our aim was to study beta-herpesvirus capsid dynamics in living cells. However, a viable, capsid tagged beta-herpesvirus was not available to track viral particle movements in living cells. Therefore, we dissected the published approaches to generate capsid-tagged fluorescent herpesviruses. Since we wanted to develop a tool that would allow to trace also emerging virus particles beginning with the first steps of capsid maturation, we chose to tag the SCP. Besides SCP, there was no successful approach reported that utilized a known component of herpesvirus procapsids. Due to the location of this protein at the outer shell of the capsid, it might allow bulky protein-tags to be added without sterically disturbing important protein-protein interactions. The SCP is essential in beta- and gamma-herpesviruses [31], [32], [35], while not essential in HSV, PRV and VZV [21], [22], [51]. All published trials concerning beta-herpesviruses were based on the direct fusion of the fluorescent protein (FP) at or into the very N-terminus of the SCPs, which resembled the strategy originally published by Desai et al. [7]. The resulting fusion proteins were non-functional and in some cases even dominant-negative, thereby preventing virus reconstitution [31], [32], [33]. Therefore, we reasoned that a free N-terminus or a sequence motif at the very N-terminus of the beta-herpesvirus SCP is essential for its function. This was further supported by an alignment of amino acid (aa) sequences of all beta-herpesvirus SCPs currently available in the databases, which showed some conservation in this region (Fig. 1A). Of most importance in this context was the discovery that MCMV encodes for a GS-linker like sequence in its SCP N-terminus (aa 10 to 34). This linker seems to separate the N-terminus into two distinct regions (Fig. 1A). We proposed that this linker gives the N-terminus the flexibility that may be required for its interaction with proteins on the capsid. A bulky fusion partner like GFP might hinder these interactions by simple sterical means, or the given flexibility of this linker sequence may not tolerate the unusual enlargement in size. Therefore, we decided to duplicate the very N-terminal region consisting of the first 34 aa and fused it, separated by an additional linker and a HA-tag, to the N-terminus of GFP. This construct was then fused to the full length SCP sequence, which includes a second, WT-copy of the N-terminal domain (Fig. 1B). As this construct consists of the first third of SCP (S), the green fluorescent protein (GFP) and the full length SCP, we named this construct S-GFP-SCP. To enable detection in different imaging setups, we also replaced the GFP coding sequence with the open reading frames of mCherry, keeping the same SCP context. To allow BAC technology-based insertion into the viral context by a simple Flp-recombinase mediated reaction, we constructed these fusion genes based on the previously published pO6-IET-gfpSCP plasmid [33]. The plasmid pOTO-S-GFP-SCP was inserted ectopically into the pSM3fr-Δm1-16-FRT BAC. This BAC codes for MCMV-Δm1-16-FRT, a virus in which the first 16 genes at the left side are deleted and an FRT-site is inserted. The deletion of these genes provided sufficient cloning capacity for the insertion of recombinant plasmids. This virus was extensively characterized and found to be indistinguishable from the wild type MCMV in tissue culture [37]. Moreover, recombinant MCMVs with similar subterminal deletions have also been reported to replicate like WT MCMV in vitro [38], [52]. As the coding sequence of the fluorescent protein (FP) in the pOTO-S-GFP-SCP vector was flanked by *Spe*I-sites, it allowed the *Spe*I-mediated removal of the FP resulting in a fusion construct that was just tagged with a HA-tag. This construct was used to control for size dependent effects of the insertion. Fig. 1B generally depicts the resulting constructs.

### Reconstitution of the Recombinant MCMVs

**Figure 2:**
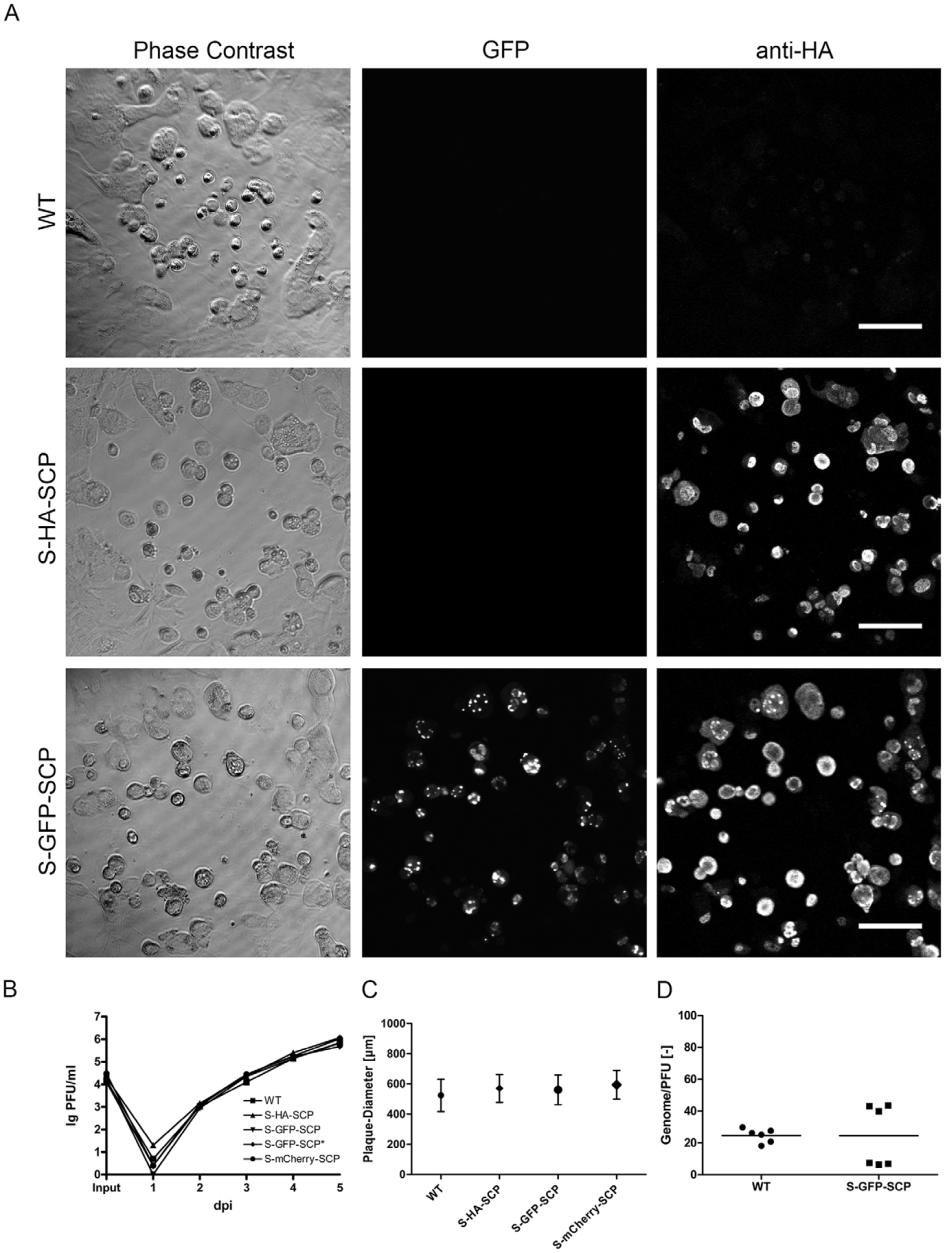
Mutants carrying tagged SCP are viable. (A) WT (WT) as well as mutant viruses coding for either HA- (S-HA-SCP) or HA-GFP-tagged wt SCP (S-GFP-SCP) were titrated on MEF cells. 4 days post infection (dpi) cells were fixed with PFA and processed for immunofluorescence against the HA-epitope. The scale bars represent 100 µm. (B) Multistep growth curve of mutant viruses used in this study in comparison to WT virus. C) Comparison of plaque diameters of simultaneously titrated WT, HA-, GFP-, and mCherry-tagged virus 4 dpi on MEF cells. (D) Genome to PFU ratio of GFP-tagged and WT virus. The ratio between genome content and titer for two independently prepared and purified virus stocks per virus was determined by titration and quantitative PCR in triplicates.

Reconstitution of the pSM3fr-Δm1-16-FRT-pOTO-S-FP-SCP constructs in NIH-3T3 cells resulted in fluorescent plaques with a distinct fluorescence pattern, which mainly localized to the nuclei of infected cells (see Fig. 2A for a representative example of the S-GFP-SCP expressing recombinant). Plaque morphologies as well as size were comparable to WT virus plaques (Fig. 2A, C). The same results were obtained for the other S-FP-SCP and the S-HA-SCP expressing recombinants (Fig. 2C). To elucidate the growth kinetics of the recombinant virus, we performed multi-step growth curves on MEF cells. All S-FP-SCP fusions used in this study as well as the S-HA-SCP mutant showed growth kinetics comparable to WT (Fig. 2B).

To compare the infectivity of the S-GFP-SCP virions to that of WT virions, we first determined the genome to PFU ratio. To this end, we prepared gradient purified virus stocks of both viruses that were routinely checked for virion integrity and purity by negative-stain electron-microscopy (EM). Subsequently, we determined the infectivity of the preparations by standard plaque assay as well as genome copies by quantitative PCR. The measured genome copies should equal the amount of DNA packaged into virus particles, as the virus stocks were treated with the DNA-degrading enzyme Benzonase during the purification process. Average values from two independent gradient purifications with simultaneous quantification of genome copies in triplicate by real time PCR, as well as plaque assays to determine the amount of PFU were compared and the resulting ratios suggested that the S-GFP-SCP expressing recombinant virus particles and WT particles show similar infectivity (23 vs. 25 genomes/PFU, respectively, see Fig. 2D). From these data we concluded that our new approach did not detectably affect viability of the recombinant MCMV.

### Characterization of FP-tagged MCMV Particles

**Figure 3:**
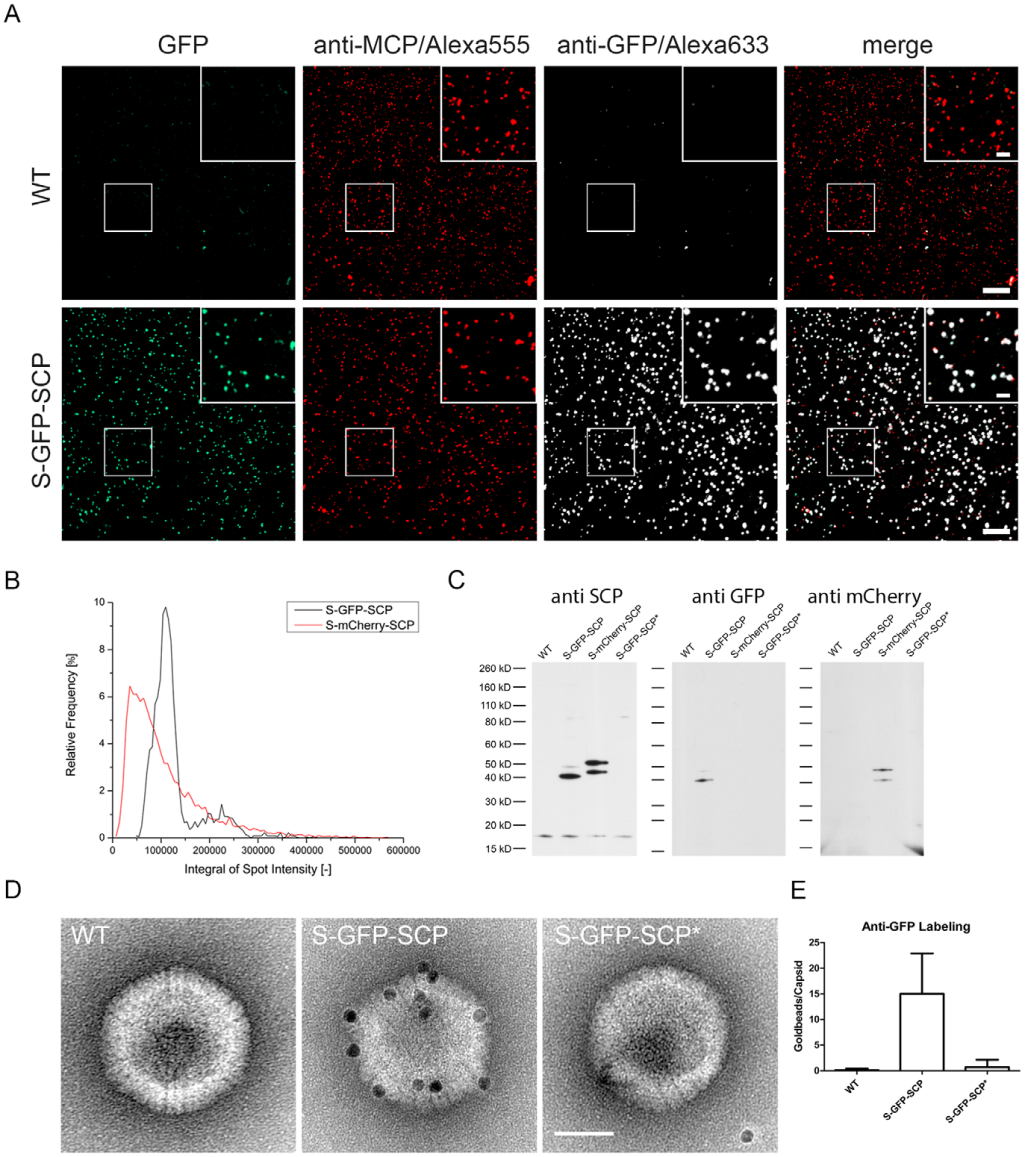
GFP-tagged SCP is incorporated specifically into virus particles. (A) Gradient purified virus particles were immobilized on fibronectin-coated cover-slips, fixed and processed for immunofluorescence. A MCP specific polyclonal serum was used as indicator of virus capsids and a GFP specific polyclonal antiserum was used to compare MCP and GFP specific signals. Direct GFP fluorescence was detected by excitation with 488 nm laser light and appropriate emission filters. Inserts depict 2x magnifications. Scale bars represent 10 µm and 2.5 µm in inserts. (B) Quantification of particle fluorescence intensity distributions. Gradient purified S-GFP-SCP or S-mCherry-SCP virus preparations were bound on Poly-Lysin coated glass-bottom dishes and fluorescent spots were recorded in 16 bit mode. Exposure times as well as the EM gain were adjusted to maximize the recorded dynamic range. The graph depicts the integrated fluorescence distributions for S-GFP-SCP (black) and S-mCherry-SCP (red). (C) Immunoblot of gradient-purified virus particles. Approximately 1*10^5^ PFU per lane of wt, (WT), S-GFP-SCP (S-GFP-SCP), S-mCherry-SCP (S-mCherry-SCP) or S-GFP-SCP* (S-GFP-SCP*) were spun down and lysed. Proteins were separated by SDS-PAGE, blotted and immunodetected with polyconal sera against SCP, GFP and mCherry. (D) Immunoelectron microscopy of purified nuclear capsids. Nuclear capsids were purified from wt (WT), S-GFP-SCP (S-GFP-SCP) or S-GFP-SCP* (S-GFP-SCP*) infected cells and immuno gold labeled with an antibody against GFP followed by protein A coupled to 10 nm gold. The scale bar indicates 50 nm. (E) Quantification of gold-labeling intensity. The amount of gold beads per capsid was counted for at least 20 views containing at least 20 capsids per condition as shown in (D). The mean as well as the standard deviation are depicted.

Next, we analyzed whether the recombinant S-GFP-SCP virus produces GFP-tagged virions. To this end, gradient-purified virions were immobilized on fibronectin-coated cover-slips and indirect immunofluorescence analysis (IF) was performed with an antiserum against the major capsid protein MCP [33] as well as with an antiserum directed against GFP (Fig. 3A). We then recorded the corresponding fluorescence signals and counted particle numbers in representative areas with the ImageJ plugin “Analyze Particles”. Colocalization was determined with the “Colocalization” command and subsequent counting of colocalized particles. We counted 2653 MCP-positive particles altogether. 91.46%+/−3.88% of them colocalized with an antiserum directed against GFP, indicating that a high amount of particles incorporated detectable amounts of S-GFP-SCP. 83.42%+/−3.64% of all MCP-positive particles could also be detected by their endogenous GFP fluorescence on a low-end confocal microscope.

Next, we wanted to elucidate if the use of different FPs to tag the MCMV SCP resulted in differing fluorescence intensity distributions as described recently for PRV mutants carrying either EGFP or mCherry tagged SCP [30]. To this end we immobilized either gradient purified EGFP or mCherry-tagged virions on Poly-L-Lysine coated cover-glass dishes and recorded particle fluorescence directly without fixation with a high dynamic range, 16 bit EMCCD camera. For the mCherry labeled virions, 8120 candidate events were initially isolated of which 5675 (∼70%) were used to determine the fluorescence distribution. For the GFP labeled virions, 2746 candidate events were initially isolated of which 2170 (∼79%) were used to determine the fluorescence distribution. The fluorescence brightness of each event was determined by integrating the best fit Gaussian model over the region of interest (Fig. 3B).

To compare the intensity histograms, we determined the full width at half maximum (FWHM) of both viruses, which is a dimensionless description of the extent of the distribution. The FWHM for S-mCherry-SCP was about 0.9*10^5^, while the FWHM for S-GFP-SCP was about 0.5*10^5^, indicating that the virus populations produced by MCMV S-GFP-SCP and MCMV S-mCherry-SCP carry comparable minimal and maximal amounts of fluorescent SCP protein. Interestingly, both distributions were consistent with multimer Gaussian distributions which was especially visible in the GFP data. It is well possible that the suggested multimers representing higher intensities are due to particle multimers forming during the preparation steps or due to virus particles having multiple capsids as we regularly observed in T-EM of infected cells (data not shown).

Next, we used Western blot analysis to visualize GFP or mCherry labeled SCP in gradient-purified virus preparations (Fig. 3C). We used three antisera, one specific for SCP, the others for either GFP or mCherry. We could detect WT SCP in the parental as well as in the GFP and mCherry tagged virus at approximately 17 kD which is a more than its calculated size of about 10 kD (Fig. 3C) which might reflect posttranslational modification. Also, by using the GFP reactive serum, we found two S-GFP-SCP and S-mCherry-SCP specific bands each running between approximately 40 and 50 kD. The upper GFP specific band was however, much weaker than the mCherry specific band. Both bands could be also detected by an antiserum reactive to SCP indicating that these two bands correspond to S-FP-SCP derivatives. These data indicated that the fusion protein is incorporated into virus particles. However, the upper GFP and mCherry specific bands were running about 8 kD higher than expected for the S-GFP-SCP or S-mCherry-SCP constructs which might reflect posttranslational modification as seen for WT SCP while the lower bands reflect the non-modified form. Moreover, the ratio between them was different when GFP and mCherry tagged virus were used, possibly reflecting differences in the amount of posttranslational processing.

### S-GFP-SCP Interacts with MCP and is Incorporated into Viral Capsids in an MCP Dependent Manner

We could show that purified virus particles are fluorescent and that this fluorescence co-localizes with an anti-MCP staining. However, this assay is not sufficient to distinguish between a specific incorporation of the S-GFP-SCP into the capsid and the unspecific incorporation of the fusion protein into virions via the tegument due to its high abundance late in infection. Therefore, we asked whether the S-GFP-SCP fusion protein could specifically engage with the capsid or a capsid constituent.

As the bulk of tegument is recruited onto capsids in the cytoplasm, we purified nuclear capsids from S-GFP-SCP infected cells to test if S-GFP-SCP binds to capsids. We then performed immunoelectron microscopy with a GFP-specific antiserum on these capsids and compared the corresponding gold-bead density with WT nuclear capsids (Fig. 3D). We could detect a mean of approximately 15 gold beads on S-GFP-SCP nuclear capsids whereas gold labeling WT nuclear capsids was not significantly higher than the background frequency arguing for a specific incorporation of S-GFP-SCP into capsids (Fig. 3D).

To further our notion, we decided to ask if S-GFP-SCP specifically interacts with a capsid constituent. To this end we used a genetic approach. It has been shown that HCMV SCP and MCP interact via a short C-terminal peptide of the SCP [53]. We therefore checked first, whether this was also true for the MCMV homologues used in our study. To this end, we deleted the corresponding peptide in the S-GFP-SCP fusion protein and tested this mutant (S-GFP-SCP*) as well as the parental S-GFP-SCP in a yeast-two-hybrid (Y2H) setup against MCP. As shown in Fig. S1, the S-GFP-SCP fusion protein interacted with MCP while the S-GFP-SCP* mutant lacking the proposed MCP-binding region did not.

Knowing that S-GFP-SCP interacts with MCMV MCP via its MCP-binding motif, we next wanted to know if the S-GFP-SCP* could be non-specifically incorporated into nuclear capsids. We therefore generated a MCMV mutant ectopically expressing the above described S-GFP-SCP* mutant, which is unable to bind to MCP and is therefore not enriched in the nuclei of infected cells (see also Fig. S2). By purifying nuclear capsids of this mutant and using them in immune-EM probing for GFP, we were able to control the interaction between the S-GFP-SCP and the MCP in the virus context. As shown in Figure 3D and 3E, gold-bead labeling of S-GFP-SCP* capsids was indistinguishable from what was seen on the WT particles clearly arguing that S-GFP-SCP* was not incorporated into nuclear capsids. Moreover, we also could not detect a GFP specific signal in a Western blot of gradient purified S-GFP-SCP* virions (Fig. 3C), showing that the S-GFP-SCP* was also not incorporated non-specifically into virions, e.g. the tegument layer. Taken together, our results indicated that S-FP-SCP fusion proteins interact with capsids and that this interaction is specific and MCP mediated.

### S-GFP-SCP and S-HA-SCP Labeled Virus Mutants are Genetically Stable

Viral genomes carrying unfavorable mutations are facing negative evolutionary pressure. The occurrence of adaptive genetic changes in a mutant locus is consequently a sign of reduced viral fitness of a given mutant. To address the genetic stability and to assess the fitness of our constructs, we passaged the S-GFP-SCP carrying virus 5 as well as 10 times on M2-10B4 cells. As controls, we used WT virus and S-HA-SCP expressing virus. We reasoned that the latter serves as a good control for insertion-size dependent effects due to the very small size of the HA-tag compared to GFP.

**Figure 4:**
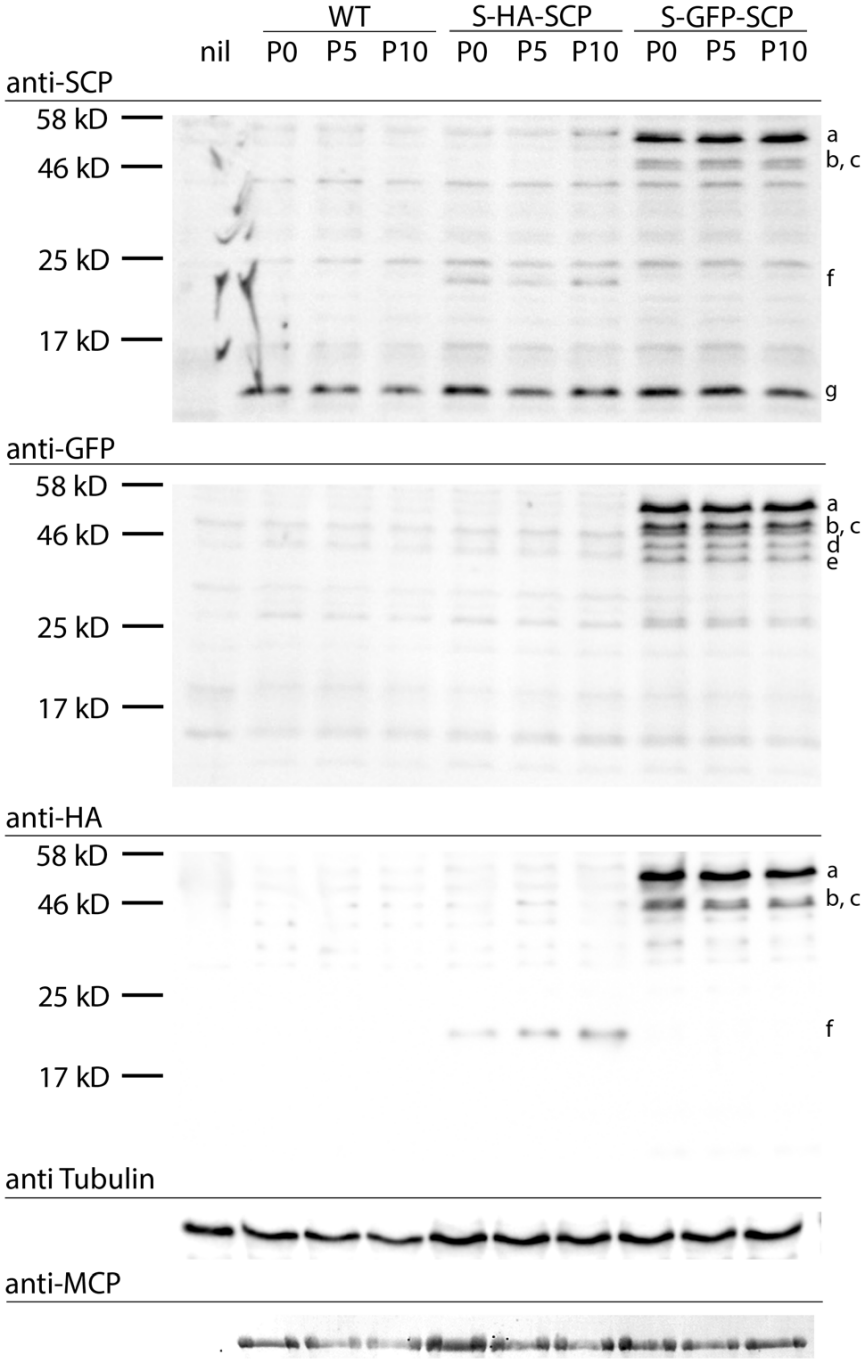
Viruses carrying labeled SCP mutants are genetically stable. Immunoblot of M2-10B4 cells infected with either wt, S-HA-SCP, or S-GFP-SCP virus derived from passages 0, 5 or 10 after reconstitution. Cells were infected at a MOI of 1, and harvested 48 hpi by lysis in total lysis buffer. Proteins were separated by SDS-PAGE, blotted and immunoprobed for GFP, SCP, HA and MCP as well as beta-Tubulin as loading controls. Lower case letters indicate discussed protein bands.

First, we wanted to know how our fusion product was expressed and which changes in protein expression occurred after extended passages. We therefore infected M2-10B4 cells with passages 0, 5, and 10 of the above mentioned viruses, lysed the cells 48 hpi and performed immunoblots specific for GFP, SCP and HA. As shown in Fig. 4, we could detect a SCP specific signal in all MCMV infected samples (band g in Fig. 4). We could also detect a S-HA-SCP specific band (band f) at approximately 22 kD instead of its predicted 12 kD with both the SCP and the HA tag specific antisera, again reflecting possible posttranslational modification. In lanes corresponding to S-GFP-SCP infected cells, we could define three bands (bands a,b,c) that stained specific for SCP, GFP and the HA-tag. The two smaller ones (bands b and c), running near the 46 kD marker band were reminiscent of the ones found in purified virions (Fig. 3C) while a slower migrating band running beneath 58 kD (band a) marker was not detected in virions. Moreover, we could detect two more bands (bands d and e) beneath 46 kD which were stained positive only by the anti-GFP antiserum. These bands might reflect SCP-FP sub-populations which represent protein degradation intermediates and/or posttranslational modifications which are present in the infected cells but not (efficiently) incorporated into the virions. However, no major changes in protein expression levels nor in the expressed protein pattern could be detected upon infection with either passage 0, 5 or passage 10 of any of the recombinant virus tested. Therefore, these data indicated that the protein expression levels of the S-HA-SCP- or S-GFP-SCP-viruses were stable for extended periods.

To further test the genetic stability of the ectopic SCP locus, we amplified the corresponding sequences by PCR from supernatants of cells infected with either passage 0, 5 or 10 of S-HA-SCP or S-GFP-SCP expressing viruses. The resulting PCR products were gel-purified, sequenced and checked for sequence alterations. Again, no alteration could be detected in either of the samples, arguing for extended genetic stability of both the S-HA-SCP and the S-GFP-SCP loci (data not shown).

### Normal Morphogenesis of S-FP-SCP Tagged Recombinant MCMV

**Figure 5:**
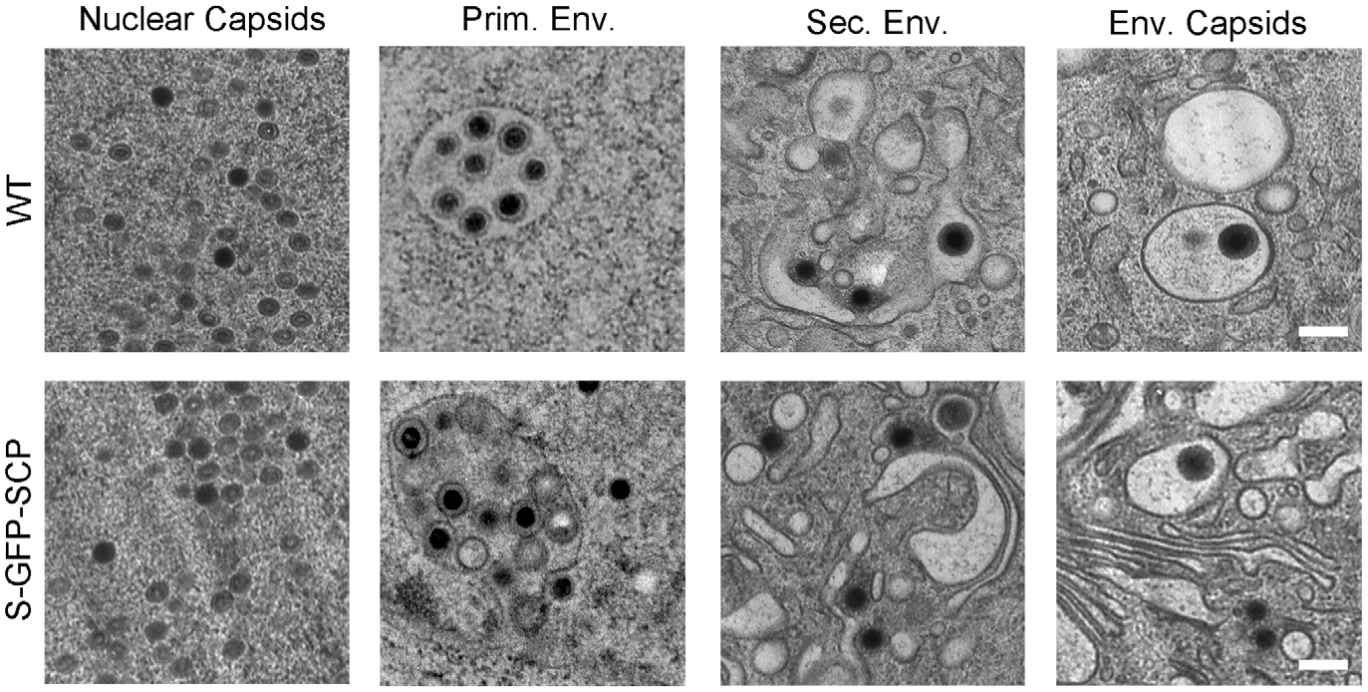
Ultrastructural assessment of S-GFP-SCP infected cells. NIH-3T3 and M2-10B4 (upper row) or M2-10B4 cells (lower row) were infected at a MOI of 0.5 and centrifugal enhancement with WT (upper row) or S-GFP-SCP labeled virus (lower row) and incubated for 48 h. Afterwards, cells were high-pressure frozen, freeze-substituted, plastic-embedded and thin-sectioned. Depicted are representative details of two independent experiments showing non-enveloped B- and C-capsids in the nucleus (first column), primary envelopment in the nucleus (2^nd^ column), non-enveloped C-capsids near cellular membranes possibly during secondary envelopment (third column), as well as enveloped capsids in the cytoplasm (right column). Scale bars indicate 200 nm.

To analyze whether virus morphogenesis is affected due to the expression of the S-GFP-SCP fusion protein, cells were infected with S-GFP-SCP virus and processed for transmission EM 48 h post infection. As depicted in Fig. 5, all major steps of virus morphogenesis e.g. capsid assembly, capsid packaging, primary envelopment in the nucleus as well as secondary envelopment appeared to be indistinguishable from WT virus in respect to phenotypic appearance and overall frequency. However, in some nuclei of S-GFP-SCP infected cells, semi-crystalline structures could be detected (data not shown). These structures mostly consisted of non-filled capsids similar to what was observed previously for a HSV VP26-mRFP virus [23] but also during WT HSV infection [54]. These data demonstrated that the major intracellular steps of the viral life cycle appeared to be normal in the fluorescently tagged MCMV recombinant virus.

**Figure 6:**
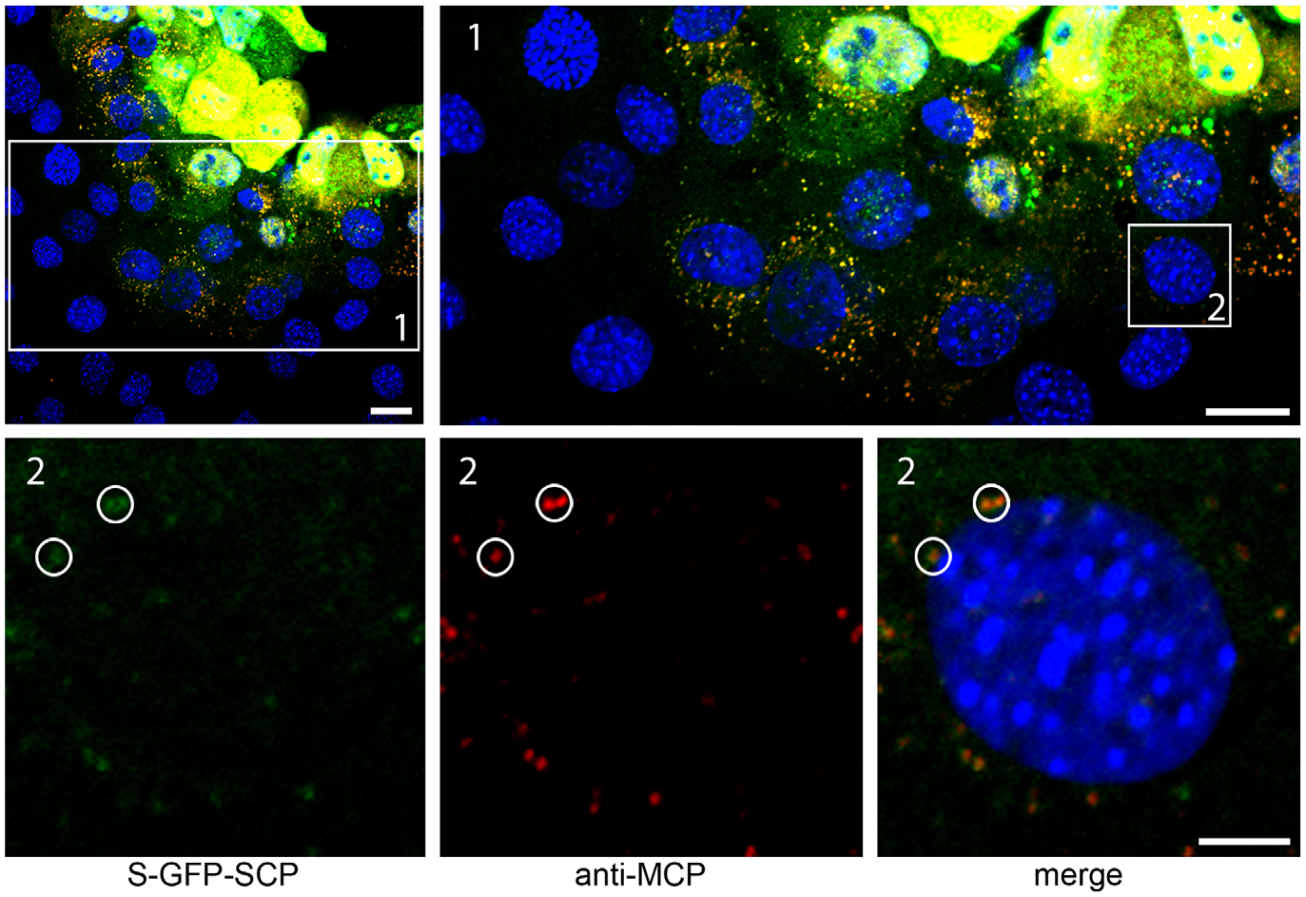
Fluorescent virus particles are spread-competent. (A) Confluent M2-10B4 cells were infected with S-GFP-SCP labeled virus on cover-slips in 24-wells with 100 PFU per well and overlaid with methyl-cellulose. 4 dpi cells were fixed and processed for immunofluorescence. MCP-specific antiserum was used to detect virus producing cells as well as single virus particles while GFP fluorescence was visualized directly. Cell nuclei were counterstained with TO-PRO-3. Inserts depict single virus particles surrounding a cell nucleus (circles) not showing any evidence for being on the late stage of infection and producing infectious particles. Scale bars indicate 20 µm in the upper row and 5 µm in the lower row.

Next, we analyzed whether fluorescent virus particles spread from cell to cell in tissue culture. NIH-3T3 cells were seeded on cover slips in 24 well plates and infected with a low MOI (100 PFU per 50000 cells). To prevent virus spread via the supernatant, we overlaid the infected cells with carboxy-methyl-cellulose as described for standard plaque assay [39]. Four days after infection, the overlay medium was removed, plaques were fixed and IF was performed. MCP was detected by a specific polyclonal antiserum and compared to S-GFP-SCP fluorescence. In addition, TO-PRO-3 was used to stain cell nuclei. As shown in Fig. 6, cells at the center of a plaque exhibited abundant MCP stain and S-GFP-SCP fluorescence (yellow) inside nuclei, indicating a late infection stage. Cells at the edge of the plaque (Fig. 6 insert 1), however, did not show any MCP or S-GFP-SCP signal in their nuclei, indicating that these cells did not started de novo synthesis of MCP and the S-GFP-SCP fusion protein. Yet, in some of these cells, we could detect fluorescent particles next to the cell’s nucleus, adjacent to the nuclear rim (Fig. 6 insert 2). These particles were positive for both GFP fluorescence and MCP. Therefore, we concluded that these particles were most likely virus particles that were transported to the nucleus of freshly infected cells. This finding suggests that labeled virions were correctly transmitted from infected to non-infected cells.

### Quantification of Cytoplasmic Movement Paths in Herpesvirus Egress

After verifying the WT virus properties of the S-GFP-SCP mutant, we aimed to use this new virus to track MCMV-infection. As tracking approaches are most easily established on virus entry scenarios at cell protrusions (due to good signal to noise ratios and planar environments) we first concentrated our work on cell protrusions (Fig. S3, Video S1 and data not shown).

**Figure 7:**
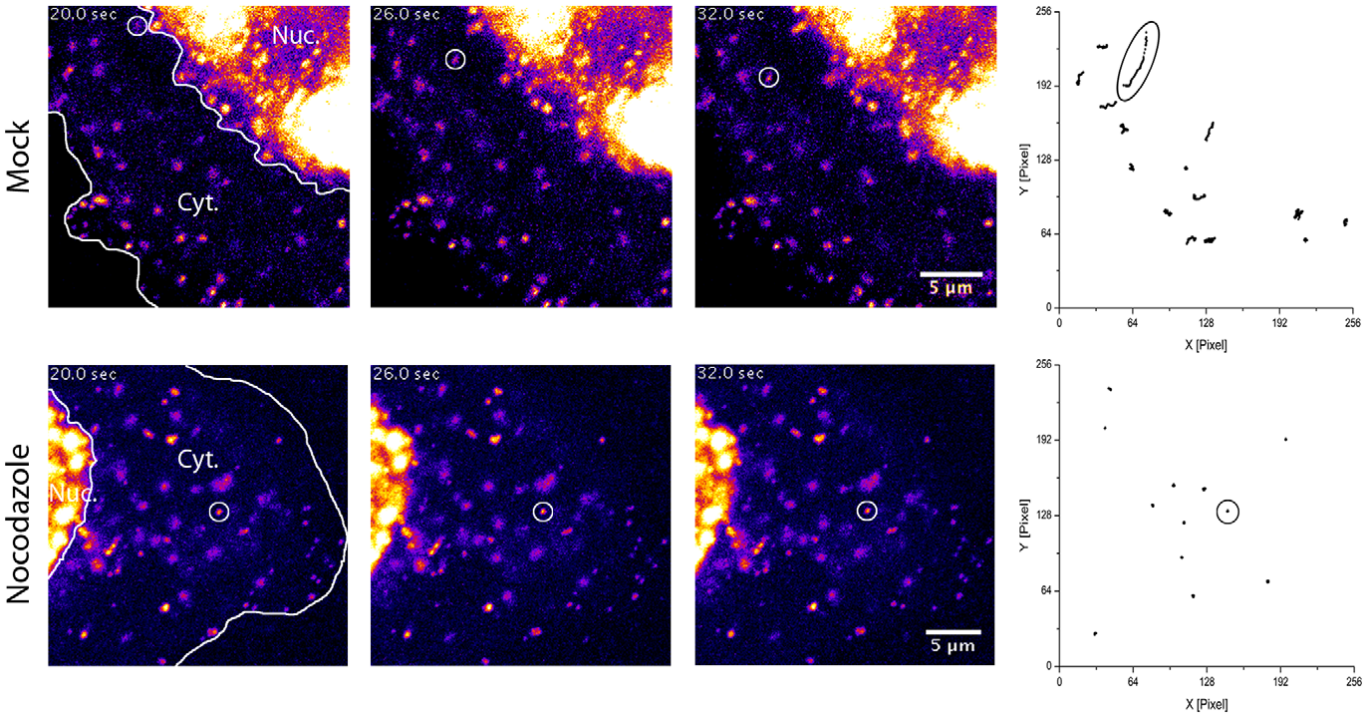
Nocodazole blocks MCMV fluorescent particle mobility. MEF cells were infected with S-mCherry-SCP for 23 h and treated with 5 µg/ml Noccodazole for 1 h or not. S-mCherry-SCP-emission in live cells recorded under environmentally controlled conditions with 5 frames per second. Fluorescence intensity is coded in false-colors from dark blue to yellow. Three frames from a time-lapse stack each recorded 6 seconds (30 frames) apart are shown for a non-treated (upper row) and Nocodazole treated cell (lower row). Lines indicate the nucleus (Nuc.) as well as the cytoplasm (Cyt.). Circles indicate the position of a fluorescent particle. The right picture depicts all quantified tracks with ellipses marking the tracks from the particles highlighted on the left.

After establishing a tracking workflow, we next wanted to know whether it was possible the quantify the intracellular movements of newly produced virus particles. In contrast to viral entry [55], this can only be solved with a genetically encoded fluorescent tag. For the following experiments, we used the mCherry-labeled MCMV variant named S-mCherry-SCP. Around 20 h post infection, the first fluorescent signals emerged in nuclei of MEFs with detection parameters suitable for live-imaging (data not shown). Around 24 hpi, fluorescent spots of varying intensity could be followed in the cytoplasm. These spots most likely represented both, non-enveloped as well as enveloped viral particles, inside of vesicles. For alpha-herpesviruses directed cytoplasmic transport of viral capsids was described to be microtubule-dependent [56]. We therefore assumed that this was also true for the beta-herpesvirus MCMV. To test whether the S-mCherry-SCP virus is suitable to quantify intracellular particle transport, we used this finding as a touchstone. We infected MEF cells at a MOI of 0.5 with MCMV encoding the S-mCherry-SCP fusion protein and dissolved the microtubule network in control cells 23 hpi by the addition of 5 µg/ml Nocodazole for 1 h and subsequently imaged cytoplasmic fluorescent signals. An antibody stain against beta-Tubulin was used to control for the effect of Nocodazole on infected cells (data not shown). The disruption of the microtubule network had a dramatic effect on the motility of cytoplasmic fluorescent spots. As shown in Fig. 7 as well as in Videos S2 and S3, cytoplasmic movements of fluorescent spots basically stopped, arguing for a microtubule dependent transport of the bulk of viral particles in the cytoplasm of MCMV infected cells.

**Figure 8:**
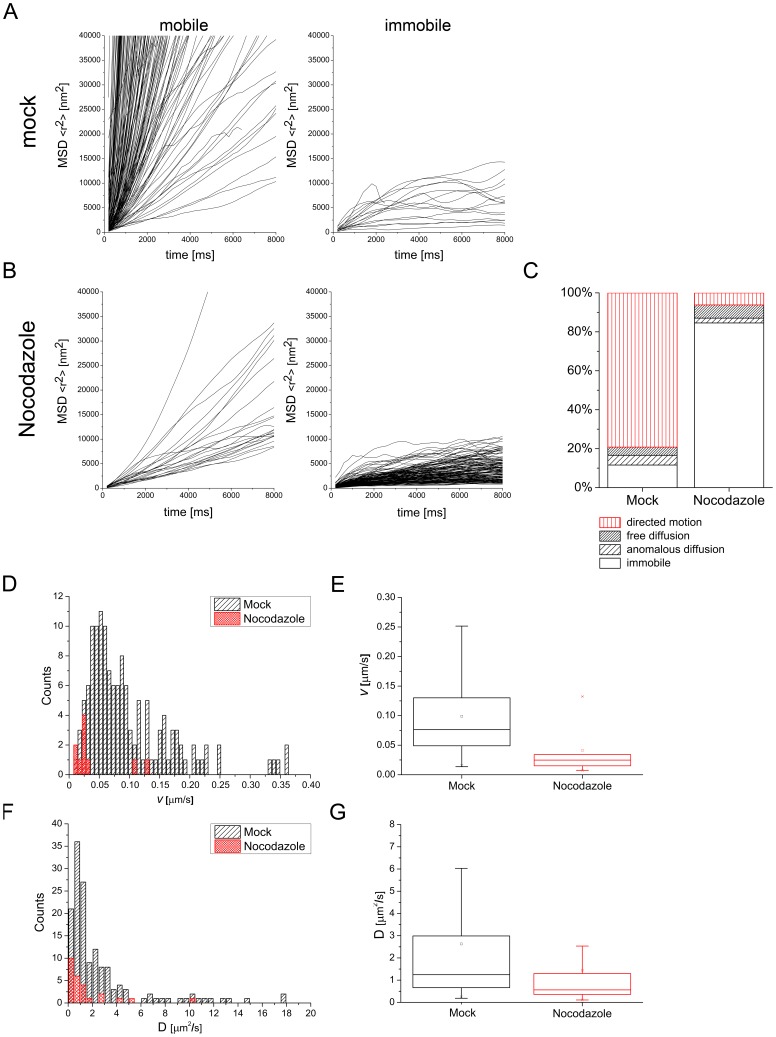
Quantification of particle tracks. (A) 180 particles from mock treated cells and were tracked and their MSDs plotted against the lag time. Particle tracks were clustered into two classes representing mobile (left diagram) and immobile particles (right diagram) depending on the MSD trend. (B) 163 tracks from Nocodazole treated cells were clustered the same way as in (A). Mobile particles were further clustered depending on which mode of diffusion they exhibited according to their fit to three different diffusion models. (C) Distribution of particle mobility classes for mock- as well as Nocodazole-treated cells depicted in percent. The data is categorized into four classes (immobile, anomalous diffusion, free diffusion, directed motion). (D) Histogram displaying the distribution of mean track velocities of all particles exhibiting directed motion. (E) The same data as in (D) summarized in a box plot with median (line) mean (square), 25^th^ to 75^th^ percentile (box) and whiskers (5^th^ to 95^th^ percentile). (F) Histogram showing the distribution of all measured diffusion coefficients. (G) Box plot of the same data shown in (F).

To quantify these cytoplasmic effects, we recorded the periphery of 7 infected mock and 7 Nocodazole treated cells at 5 frames per second and subsequently tracked randomly most identifiable fluorescent particles (180 particles in mock-treated cells/163 particles in Nocodazole-treated cells). We then calculated their MSD curves by determining the mean square displacements (MSD) and plotting them against the lag time. The resulting curves were clustered into two classes. The first class included all mobile particles and the second all immobile particles. We discerned between these two classes by analyzing their MSD curves and taking the experimental positioning accuracy of the used tracking algorithm into account. We determined an experimental positioning error of around 30 nm per axis which translates into approximately 14.500 nm^2^ in our MSD plots. All curves that did not exceed this value were classified as immobile, because their change in position during acquisition was less than the experimental localization accuracy of 30 nm per axis. In contrast, all particles that exceeded this value were classified as mobile (Fig. 8A, B). According to this quantification, 88.4% of particles in mock treated cells were mobile. In contrast, 84.6% of particles in Nocodazole treated cells were immobile. Surprisingly, 15.4% of the particles in Nocodazole treated cells were still mobile after 1 h incubation with 5 µg/ml Nocodazole (Fig. 8C).

The mode of diffusion was determined by calculating the mean square displacements (MSD) and plotting them against the lag time. To analyze the properties of the mobile populations further, we fitted the corresponding MSD curves in a two-step process according to three diffusion models. These models were anomalous diffusion (MSD  = 4D*t^α^*), free diffusion (MSD  = 4D*t*) and directed motion with diffusion (MSD  = 4D*t+* (*vt)^ 2^*) (for details see materials and methods).

79.2% of mobile particle tracks in mock treated cells could be fitted with the function MSD  = 4D*t+(vt)^2^* compared to 6.2% in Nocodazole-treated cells, indicating that a small fraction of particles in Nocodazole-treated cells exhibited directed motion, hinting to a possibly Nocodazole-resistant transport mode.

Interestingly, Nocodazole treatment did not strongly influence the amount of particles diffusing freely (fit with MSD  = 4D*t*) or in an anomalous fashion (fit with MSD  = 4D*t^α^*) (Fig. 8C). Together with the strong increase of immobile particles under Nocodazole treatment this findings might suggest that viral particles were either trapped in a very small molecular cage or fixed to some molecular anchor after Nocodazole treatment.

As we fitted the mobile fraction of particles that exhibited directed movement with MSD  = 4D*t+(vt)^2^*, we could directly determine the overall mean velocity of a given particle track without its diffusion component. As shown in Fig. 8D and E, mean track velocities (over the complete acquisition time of approximately 20 sec) in mock treated cells had a mean of 0.101±0.073 µm/sec. In Nocodazole treated cells however, particles that exhibited directed movement had a mean, mean track velocity of only 0.041±0.042 µm/sec. This finding indicated that high mean track velocities were mostly based on a Nocodazole-sensitive, and therefore most likely, microtubule-associated transport mode.

The second parameter we could determine was the diffusion coefficient D. It was calculated for all mobile particles and the median in mock-treated cells was 1.24·10^−8^ cm^2^/s while 0.56·10^−8^ cm^2^/s in Nocodazole-treated cells (Fig. 8F,G). Interestingly, the distribution width of D in mock treated cells was higher than expected for a monodispersed particle suspension (Fig. 8F). D is inversely proportional to the hydrodynamic radius of a particle, as long as all other variables like temperature and the viscosity of the medium are not changed (D  =  kT/6πηr). Therefore, the observed distribution of D could be an indicator for a wider range of tracked particle diameters.

## Discussion

In this study, we report on beta-herpesviruses that are genetically tagged at their capsid by FPs. These recombinant viruses express an additional copy of engineered SCP in *cis* that carries the FP at an internal position joined via two flexible linkers. This second FP-tagged SCP gene is expressed under the control of its own promoter to maintain physiological levels of expression. This strategy resulted in production of capsids harboring a mixture of FP-tagged WT SCP. The presence of the WT SCP seems to be important, as a similar recombinant BAC lacking the endogenous SCP was not viable (data not shown). This phenomenon that a WT allele completely compensates a non-functional mutation present on a separate allele is well known in herpesvirus genetics and was utilized to map temperature-sensitive mutants in the past [57]. The herein described case is similar, but they differ in that the two alleles are in one genome. Why the WT SCP needed is unclear. One explanation are sterical constraints as the bulky fluorescent protein might be perhaps too big to fit six times onto a capsid hexon, while a mixture of unlabeled and labeled SCPs does. Another explanation is that the labeled variant cannot engage in essential interactions with neighboring proteins, but as not all molecules need to engage in this interaction, the unlabeled proportion of SCP molecules might be sufficient to compensate this deficit. Yet, the recombinant MCMV carrying S-HA-SCP was viable even after deletion of the native SCP locus (data not shown) arguing for tag-size dependent deficiency of the presented S-FP-SCPs. This size-dependence may allow the tagging of SCPs at the native locus utilizing for example the recently published smaller LOV domain-based, new fluorescent tags like iLov [58] using our design.

The S-GFP-SCP fusion protein was clearly incorporated into virus particles, which is due to its interaction with MCP. However, we cannot exclude that the S-FP-SCP fusion proteins lack other binding functions that have to be substituted by endogenous SCP. Therefore, although our mutant fusion protein is not a substitute for WT SCP, it is not inhibitory as shown virus growth kinetics, plaque diameter and genome number to PFU ratios. The WT-virus like properties of the new recombinant FP tagged MCMVs are an important advance, as a quite similar GFP-SCP fusion developed earlier in our laboratory showed a strong dominant negative effect on virus growth when expressed in *cis* from an inducible locus [59]. The difference between these two fusions is essentially the duplication of the first 34 aa that are added to the N-terminus of the fluorescent protein. These 34 aa might engage in important interactions or provide additional flexibility that discerns the S-GFP-SCP fusion from its dominant negative ancestor gfp-SCP.

Recently, it was shown that PRV mutants carrying a GFP tagged SCP exhibit a much wider intensity distribution than an equivalent mutant carrying mRFP instead of GFP. [30]. In the here-described mutants, the FWHMs were quite similar, with an about two-fold difference between GFP and mCherry (an mRFP variant). In our case however, the mCherry-tagged mutant showed the higher value compared to the GFP-tagged variant. Interestingly, we found evidence for a multimer Gaussian distribution. This might be due to virus particles having multiple capsids as we regularly observed in T-EM of infected cells (data not shown) or due to aggregation of particles during the purification procedure which is necessary to remove cellular debris.

We next tested for phenotypic changes in the ultrastructure of infected cells and could not find obvious morphological differences except for some cells in a late stage of infection that showed parachrystalline arrays of what seemed to be B-capsids. This phenomenon is also described for HSV-1 carrying an mRFP-tagged SCP as well as for WT HSV-1 infection [23], [54].

The quantification of capsid labeling intensity as well as the proportion of labeled particles revealed that most particles are labeled to an extent that permitted particle tracking.

We also tried to tag the SCP of HCMV by the same strategy and this resulted in a strongly attenuated recombinant virus. These data indicated that the procedure was merely correct but that fine-tuning of the labeling approach is necessary for each herpesvirus species. Probably, the size of the inserted tag is crucial. Also, there might be a difference in oversize tolerance in the different herpesvirus species.

Next, we tested purified virus preparations of MCMV S-GFP-SCP in an entry assay and could detect particle attachment to cell protrusions and trafficking along those. We were able to establish a tracking workflow with few particles that resulted in a very accurate localization of single virus particles (45±19 nm) (see Fig. S3). Tracking extracellular viral particles provides an environment with low background and low particle densities and is ideal to establish a tracking workflow. But viral entry in general can be, at least with non-enveloped viruses, also followed with chemically labeled virions [55]. The huge advantage of a virus genetically encoding fluorescent markers is the possibility to follow the fate of newly produced intracellular viral particles. Particle movement in the cytoplasm during virus egress, however, happens in an environment characterized by high background noise, high particle counts and a reduced cell cross section due to virus induced cell rounding. We tracked particles shortly after they appeared first in the cytoplasm and tracked their motility. As negative-control we used infected cells treated with the microtubule depolymerizing drug Nocodazole. By using a sophisticated fitting approach, we could cluster the obtained particle tracks into four classes representing immobile particles and mobile particles exhibiting either anomalous diffusion, free diffusion or directed motion. We found that Nocodazole treatment reduced the fraction of mobile particles drastically from approximately 88% to 15%. This effect was mainly due to the reduction of particles exhibiting directed motion (79% to 6%), while the proportion of particles exhibiting free or anomalous diffusion did not change strongly. This was anticipated, as it was shown before that viral cytoplasmic transport is mainly microtubule-mediated. However it was surprising, that the dissociation of microtubules by Nocodazole did not increase the proportion of free or anomalous diffusing particles much (4.4% to 6.8% and 5.2% to 2.5% respectively).

This finding might indicate that particles are still bound to microtubule fragments after Nocodazole treatment as the tracking data indicated a very large proportion of particles that did not move more than the localization precision of 30 nm (classified as immobile). Yet, other mechanisms for immobilization of capsids, such as entrapment in a molecular cage or fixation to a molecular anchor, are also possible.

Remarkably, still around 6% of particle tracks from Nocodazole-treated cells could be fitted according to a directed motion model. Two explanations seem plausible. First, the remaining movement upon treatment with Nocodazole may be caused by the presence of stabilized Nocodazole resistant microtubules, similar to those which have been described for HSV-1 infections [60]. Alternatively, the observed Nocodazole-resistant movement is based on actin filaments, a hypothesis that is underlined by the recent report describing the dependence of HSV-1 virion secretion on Myosin V [61]. However, to specifically address this question, future experiments are needed.

Instead of a focused distribution of the diffusion coefficient as anticipated for a monodisperse solution of particles, we found a wider distribution of D. Assuming a uniform cytoplasmic viscosity this finding would translate into a non-uniform distribution of vesicle sizes used by the virus to transport particles. A simple explanation for the wider distribution of D could be that it reflects to the size difference between enveloped and naked capsids. In addition, we could observe a great difference in vesicle sizes for virus containing vesicles in transmission EM (Fig. 5 and data not shown). The sum of these interpretations might translate to the observed phenotype. Moreover, it demonstrates the power of single-particle tracking to probe the environment of the measured particles without the need of extensive co-labeling.

We also calculated mean track velocities from particle tracks which could be fitted according to a model for directed movement. Notably, this mean track velocities were much smaller than what is reported for HCMV [15]. This might have several reasons apart from intrinsic differences between HCMV and MCMV: First, we randomly chose particles that were technically possible to track and did not select for mobile particles. Therefore, our mean step velocities had to be smaller as more slowly moving particles were included. In addition, we used a different tracking and calculation approach. The mean track velocities used here, display the mean velocity a given particle exhibits over the whole measured track. As we did not select for fast transport processes during tracking nor used track segmentation as shown in Fig. S3, our mean track velocities had to be smaller than velocities from “hand-picked” (sub-)tracks. The approach taken here should be therefore much better suited to approximate a realistic mean transport velocity with which cytoplasmic herpes particles are transported over longer time spans.

Up to now, we did not quantify the cytoplasmic motility of other similarly tagged viruses. Future studies are aimed to discern enveloped from non-enveloped particles (e.g. with an additional glycoprotein-FP fusion) and to study the direction of transport to produce a more detailed picture of cytoplasmic transport processes during MCMV egress.

Altogether, we were able to generate the first viable and stable recombinant beta-herpesvirus that produces fluorescently labeled capsids. In comparison to the widely used HSV-1 and PRV capsid-tagged mutants, the here described recombinant did not show any growth defect in cell culture and exhibited biological features comparable to their parental WT virus [5], [34]. All tested recombinants produced fluorescent virus capsids with an intensity sufficient to track single particles over extended periods in live-cell microscopy. The analysis of intracellular particle transport during MCMV egress, for the first time with subpixel-spatial- and high temporal- resolution in the herpesvirus field, enabled us to characterize the cytoplasmic transport of a beta-herpesvirus with high detail.

Future studies are entitled to transfer the herein described approach to label a gamma-herpesvirus which would enable the comparative study of alpha-, beta- and gamma-herpesvirus capsid dynamics.

## Supporting Information

Figure S1 **(in support of** **Fig. 3** ) Schematic diagram summarizing the results from a yeast two-hybrid assay, probing the interaction between MCP and S-GFP-SCP or S-GFP-SCP*. The S-GFP-SCP* mutant lacks the last 14 aa at the C-terminus which are predicted to interact with MCP. As a control, empty bait and prey plasmids were used (ctrl). White squares indicate no growth on selective agar and a failure of interaction. Black squares indicate growth on selective agar and an interaction.
(TIF)

Figure S2 **(in support of** **Fig. 3** ) MEFs were seeded in 8-well plastic slides and infected with 100 PFU of virus/per well expressing ectopically either S-GFP-SCP (top) or a S-GFP-SCP fusion protein lacking its proposed MCP-interaction peptide (S-GFP-SCP*, bottom). Cells were overlaid with methyl-cellulose after infection and fixed and processed for immunofluorescence 4 dpi. GFP fluorescence was visualized directly while the cytoplasm and cell nuclei were counterstained with a high concentration of TO-PRO-3, thereby staining whole cells but still indicating the cell nuclei. The mutant lacking its MCP interacting peptide is localized throughout the cyto- and nucleoplasm. Scale bars indicate 10 µm.
(TIF)

Figure S3 **(in support of** **Fig. 7** **and** **8** **) Tracing of extracellular particle trafficking.** M2-10B4 cells were seeded at low density on glass-bottomed culture dishes and infected at a MOI of 100 with gradient purified and EM-controlled S-GFP-SCP virus stock. Directly after infection, live imaging with 488 nm laser excitation as well as differential interference contrast (DIC) was started in an environmentally controlled chamber. Virus particles attached to cell protrusions were identified by their fluorescence and recorded with 1.8 frames per second. (A) The changing positions of two fluorescent particles on a cell protrusion are depicted over time (circles). Numbers indicate individual particles. (B) Tracks of particle 1 and particle 2 (insert). No positions for the particles 1 and 2 could be obtained where particle tracks overlapped; therefore each particle track is divided in two parts (both are black for particle 1, red and black for particle 2). Manual track separation was done by choosing sub-trajectories in which the particles clearly exhibit long-distance movements. Each obtained sub-trajectory was then analyzed individually by computing their MSDs and fitting the resulting MSDs plots according to the models stated for Fig. 8. The 19 manually chosen subtracks in which particles clearly showed long distance movements are indicated by color overlays. The direction of movement is indicated by arrows. (C) Histogram displaying the distribution of measured mean track velocities for all used subtracks in which particles showed long-distance movements. (D) Histogram depicting the distribution of measured diffusion coefficients for the same subtracks as in (C). We determine the overall mean track velocity (v) and diffusion coefficient (D) from all subtracks. For the two selected particles the mean track velocity of the subtracks was 0.10±0.05 µm/sec and the mean of D was 5.87±3.3 µm^2^/sec, which indicated a high mobility (D).
(TIF)

Table S1 **Summary of primers used in this study.**
(PDF)

Video S1 **(in support of** **Fig. 7** **and Figure S3) Tracing of extracellular MCMV trafficking.** M2-10B4 cells were seeded at low density on glass-bottomed culture dishes and infected at a MOI of 100 with gradient purified S-GFP-SCP virus stock. Directly after infection fluorescence live cell imaging with 488 nm laser excitation in parallel with differential interference contrast (DIC) microscopy was started in an environmentally controlled chamber. Virus particles attached to cell protrusions were identified by their fluorescence and recorded with 1.8 frames per second. The video is an overlay of the DIC and GFP channels.
(AVI)

Video S2 **(In support of** **Fig. 7** **) Nocodazole blocks MCMV fluorescent particle mobility.** MEF cells were infected with S-mCherry-SCP for 23 h and treated with 5 µg/ml Nocodazole for 1 h. Imaging and presentation conditions were the same as for video S3.
(AVI)

Video S3 **(In support of** **Fig. 7** **) Mobility of fluorescent MCMV particles.** MEF cells were infected with S-mCherry-SCP for 24 h. Afterwards S-mCherry-SCP-emission was recorded in live cells under environmentally controlled conditions with 5 frames per second. Fluorescence intensity is coded in false-colors from dark blue to yellow.
(AVI)
