# Supplementary figures and images for: A Beta-Herpesvirus with Fluorescent Capsids to Study Transport in Living Cells
Source: PLoS One. 2012 Jul 11;7(7):e40585. doi: 10.1371/journal.pone.0040585 (PMC3394720; doi:10.1371/journal.pone.0040585)

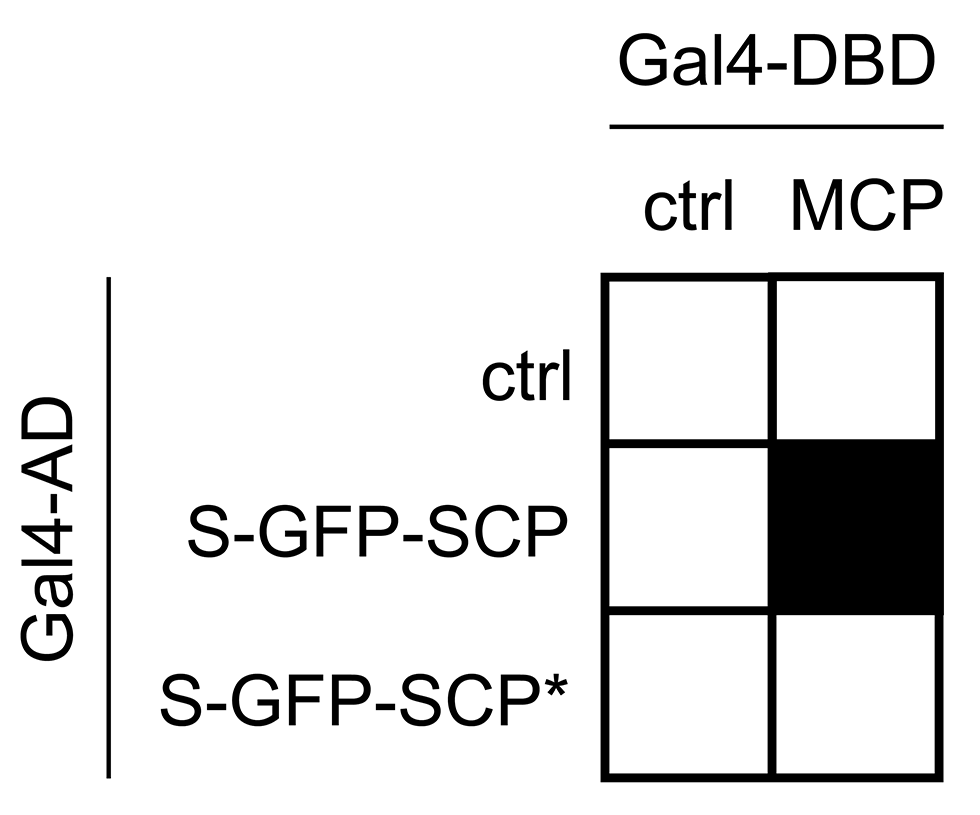

Supplement: Figure S1 — (in support of Fig. 3 ) Schematic diagram summarizing the results from a yeast two-hybrid assay, probing the interaction between MCP and S-GFP-SCP or S-GFP-SCP*. The S-GFP-SCP* mutant lacks the last 14 aa at the C-terminus which are predicted to interact with MCP. As a control, empty bait and prey plasmids were used (ctrl). White squares indicate no growth on selective agar and a failure of interaction. Black squares indicate growth on selective agar and an interaction. (TIF) [file pone.0040585.s001.tif]

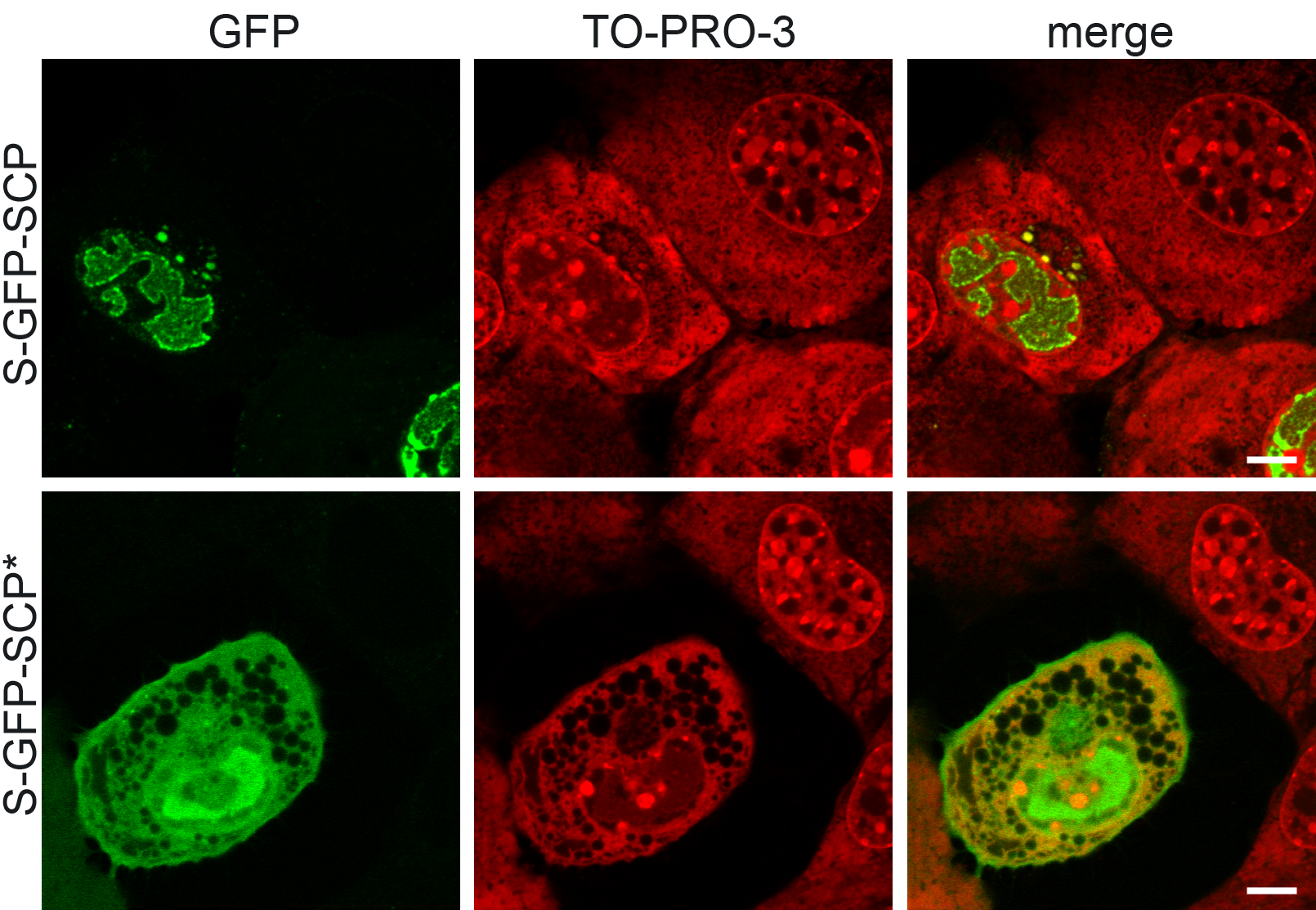

Supplement: Figure S2 — (in support of Fig. 3 ) MEFs were seeded in 8-well plastic slides and infected with 100 PFU of virus/per well expressing ectopically either S-GFP-SCP (top) or a S-GFP-SCP fusion protein lacking its proposed MCP-interaction peptide (S-GFP-SCP*, bottom). Cells were overlaid with methyl-cellulose after infection and fixed and processed for immunofluorescence 4 dpi. GFP fluorescence was visualized directly while the cytoplasm and cell nuclei were counterstained with a high concentration of TO-PRO-3, thereby staining whole cells but still indicating the cell nuclei. The mutant lacking its MCP interacting peptide is localized throughout the cyto- and nucleoplasm. Scale bars indicate 10 µm. (TIF) [file pone.0040585.s002.tif]

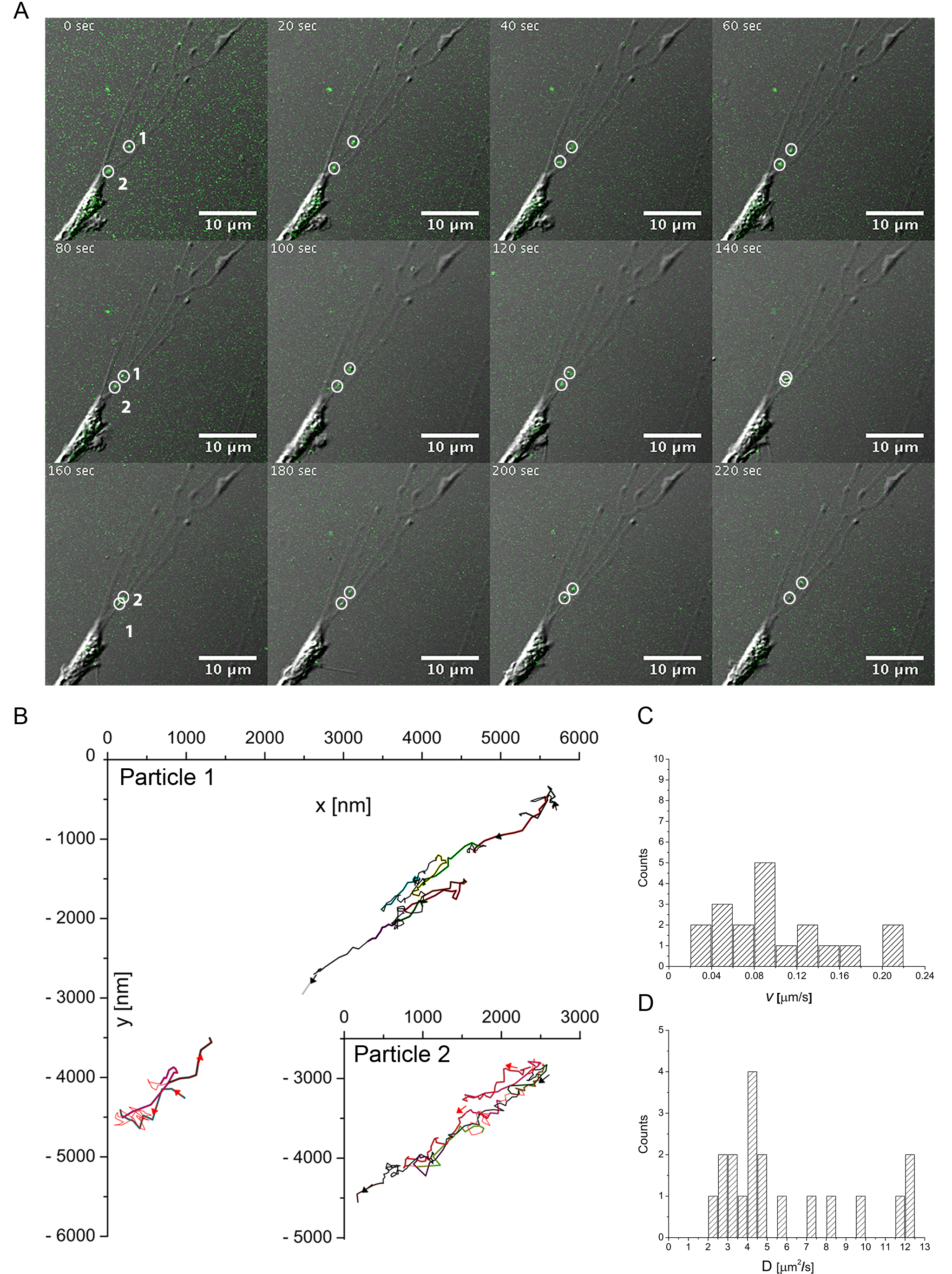

Supplement: Figure S3 — (in support of Fig. 7 and 8 ) Tracing of extracellular particle trafficking. M2-10B4 cells were seeded at low density on glass-bottomed culture dishes and infected at a MOI of 100 with gradient purified and EM-controlled S-GFP-SCP virus stock. Directly after infection, live imaging with 488 nm laser excitation as well as differential interference contrast (DIC) was started in an environmentally controlled chamber. Virus particles attached to cell protrusions were identified by their fluorescence and recorded with 1.8 frames per second. (A) The changing positions of two fluorescent particles on a cell protrusion are depicted over time (circles). Numbers indicate individual particles. (B) Tracks of particle 1 and particle 2 (insert). No positions for the particles 1 and 2 could be obtained where particle tracks overlapped; therefore each particle track is divided in two parts (both are black for particle 1, red and black for particle 2). Manual track separation was done by choosing sub-trajectories in which the particles clearly exhibit long-distance movements. Each obtained sub-trajectory was then analyzed individually by computing their MSDs and fitting the resulting MSDs plots according to the models stated for Fig. 8. The 19 manually chosen subtracks in which particles clearly showed long distance movements are indicated by color overlays. The direction of movement is indicated by arrows. (C) Histogram displaying the distribution of measured mean track velocities for all used subtracks in which particles showed long-distance movements. (D) Histogram depicting the distribution of measured diffusion coefficients for the same subtracks as in (C). We determine the overall mean track velocity (v) and diffusion coefficient (D) from all subtracks. For the two selected particles the mean track velocity of the subtracks was 0.10±0.05 µm/sec and the mean of D was 5.87±3.3 µm2/sec, which indicated a high mobility (D). (TIF) [file pone.0040585.s003.tif]
