# Supplementary material for: A Beta-Herpesvirus with Fluorescent Capsids to Study Transport in Living Cells
Source: PLoS One. 2012 Jul 11;7(7):e40585. doi: 10.1371/journal.pone.0040585 (PMC3394720; doi:10.1371/journal.pone.0040585)
Supplement: Table S1 — Summary of primers used in this study. (PDF) [file pone.0040585.s004.pdf]

## Table S1. Primer sequences

| Primer | Sequence[a,b] |
|---|---|
| MCMV-for[c] | ATCATCCGTTGCATCTCGTTG |
| MCMV-rev[c] | CGCCATCTGTATCCGTCCAT |
| M54-as[c] | [FAM]-AACGTACATCGCTCTCTGCTGGCCG-6-carboxytetramethylrhodamine [TAMRA] |
| SCPPfor | GTGTGATATCGTCGTGCTGCGCGAGTCATCC |
| SCPPrev | GTGTCTTAAGACTAGT*GGCGTAGTCGGGCACGTCGTAGGGGTA*GCTGCTGCCGCCGCTTCCTCC |
| SP-*Afl*II-FP-*Bsr*GI | GTGACTCTTAAGGCCACCATGGTGAGCAAGGGCGAGGA |
| ASP-*Afl*II-FP-*Bsr*GI | CACAGTTGTACAGCTCGTCCATGCCGCCGGTGG |
| SP-MCP-binding-negative | ATCGTGCAGCTGGACCTTTAAGACACACATACAGAAAAATAAA |
| ASP-MCP-binding-negative | AGTCACGGGCCCACATGTGCGAGGCC |
| attB1-S-GFP-SCP-attB2-SP | GGGGACAAGTTTGTACAAAAAAGCAGGCTTACTAGCTCTCTCTACTTTA |
| attB1-S-GFP-SCP-binding-negative-No-Stop-attB2-ASP | GGGGACCACTTTGTACAAGAAAGCTGGGTAAAGGTCCAGCTGGTAGGCGAG |
| attB1-SCP-S-GFP-SCP-no-stop-attB2-ASP | GGGGACCACTTTGTACAAGAAAGCTGGGTATTGTATGACGGTGGCTTT |

[a] Underlined nucleotides: cleavage sites for restriction enzymes

[b] Italic nucleotides: coding sequences for protein tags

[c] Scrivano, L., J. Esterlechner, H. Mühlbach, N. Ettischer, C. Hagen, K. Grünewald, C. A. Mohr, Z. Ruzsics, U. Koszinowski, and B. Adler. 2010. The m74 gene product of murine cytomegalovirus (MCMV) is a functional homolog of human CMV gO and determines the entry pathway of MCMV. J. Virol. 84:4469–4480.
